# Seeking Rules Governing Mixed Molecular Crystallization

**DOI:** 10.1021/acs.cgd.2c00992

**Published:** 2022-12-15

**Authors:** Norbert M. Villeneuve, Joshua Dickman, Thierry Maris, Graeme M. Day, James D. Wuest

**Affiliations:** † Département de Chimie, Université de Montréal, Montréal, Québec H2V 0B3, Canada; ‡ School of Chemistry, University of Southampton, University Road, Southampton SO17 1BJ, United Kingdom

## Abstract

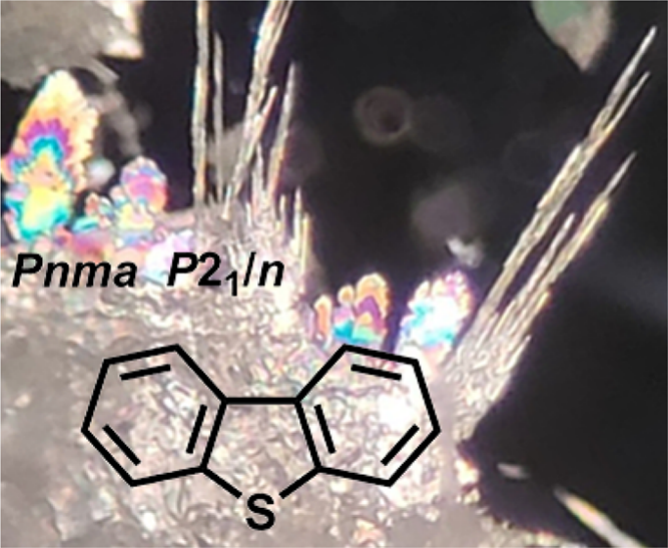

Mixed crystals result when components of the structure are randomly replaced by analogues in ratios that can be varied continuously over certain ranges. Mixed crystals are useful because their properties can be adjusted by increments, simply by altering the ratio of components. Unfortunately, no clear rules exist to predict when two compounds are similar enough to form mixed crystals containing substantial amounts of both. To gain further understanding, we have used single-crystal X-ray diffraction, computational methods, and other tools to study mixed crystallizations within a selected set of structurally related compounds. This work has allowed us to begin to clarify the rules governing the phenomenon by showing that mixed crystals can have compositions and properties that vary continuously over wide ranges, even when the individual components do not normally crystallize in the same way. Moreover, close agreement of the results of our experiments and computational modeling demonstrates that reliable predictions about mixed crystallization can be made, despite the complexity of the phenomenon.

## Abstract 2

Suitably designed computational methods can be used to assess the feasibility of mixed crystallization, provide a deeper understanding of the phenomenon, and help lead to the discovery of new solid forms.

## Introduction

Using crystallization to purify compounds predates recorded history.^1^ The simplicity, scope, and effectiveness of the process continue to make crystallization an indispensable tool. The periodic structure of crystals reflects a high degree of molecular recognition, which promotes growth by the addition of identical components and disfavors the incorporation of foreign substances. Nevertheless, crystallization does not necessarily yield compounds in pure forms, and many other outcomes are possible.^2^ For example, hydrates or other solvates can result when the primary components of crystals interact with solvents or simply do not pack efficiently by themselves, leaving space for including guests.^3−6^ Alternatively, two or more different solids can cocrystallize to form composite structures in which the components are present in a defined ratio and occupy specific sites in the lattice.^7,8^ In addition, mixed crystals (also called solid solutions) can result when structurally related compounds are accommodated at essentially random sites in the lattice in quantities that can be varied continuously over certain ranges.^9,10^ Impurities can also be incorporated adventitiously in crystals when growth occurs rapidly and material near the expanding surface is occluded.

Because crystallization is useful and because the potential outcomes have fascinating diversity, the purposeful growth of crystals in the presence of foreign substances has attracted scientific interest for centuries. For example, Robert Boyle studied the phenomenon and published the following observation in his treatise on *The Origins of Forms and Qualities* almost four centuries ago: “Notwithstanding the regular and exquisite figures of some salts, they may, by the addition of other bodies, be brought to constitute crystals of very different yet curious shapes.”^11^ Modern studies of adsorption on growing crystals have provided an atomically detailed understanding of how additives can change crystalline morphology by binding reversibly to specific faces, interfering selectively with further adsorption, and allowing unimpeded growth elsewhere.^12,13^

Reversible adsorption of this type can alter the morphology of crystals without necessarily introducing impurities. However, it is also possible for suitable additives to bind to surfaces in ways that do not interfere substantially with further growth. In such cases, the additives become incorporated as impurities. This gives rise to the phenomenon of doping, when the levels of impurity are low, or to the formation of mixed crystals, in which higher amounts of additives are present. Obviously, additives of this type must closely resemble the other components of the lattice to allow all species to be accommodated within a single ordered structure.

Unfortunately, no clear rules exist to predict when two compounds that are not merely isotopologues are similar enough to form mixed crystals containing substantial amounts of both. Conversely, it is not generally known in advance if crystals obtained from particular mixtures of compounds will be essentially free of contaminants. Knowledge of this type is not merely of academic interest. In processes that use crystallization for purification, the crude material often contains closely related compounds resulting from the method of synthesis. In favorable cases, impurities will be excluded when the compound of interest is crystallized; in other cases, however, the contaminants will be readily incorporated in mixed crystals. The outcome is typically determined empirically, but deeper understanding of mixed crystallization may reveal in advance whether the formation of impure crystals is likely or improbable. Methods of synthesis can thereby be chosen to prevent the formation of potentially troublesome contaminants. In such ways, crystallization can be made more reliable as a method of purification, and costlier alternatives such as chromatographic separations can be avoided. Moreover, when the goal is to introduce impurities intentionally to form doped crystalline materials or to make mixed crystals with continuously variable compositions and properties, the capacity of the primary component to accommodate other species must be assessed. Acquiring this information by experimentation is slow, and the discovery of new materials needs to be accelerated by developing effective tools for predicting when substitutions within a crystal lattice are feasible.

Current understanding of principles governing the mixed crystallization of organic and inorganic compounds is based largely on work carried out decades ago by Kitaigorodsky and co-workers, which has not been subjected to extensive reexamination.^14,15^ These pioneering studies suggested that a series of mixed crystals with continuously variable compositions in all proportions can only be obtained when the components are similar enough to crystallize isostructurally.^14^ However, more recent work has challenged this notion and shown that mixed crystals with a wide range of compositions can be formed from pairs that are not known to have a close crystallographic relationship.^16−30^ These recent advances, which include the discovery that mixed crystals can be effective seeds for inducing crystallization of the individual components,^28^ have made mixed crystallization an exciting area of research.

To probe the phenomenon in greater detail, we have studied the mixed crystallization of a carefully selected set of four compounds: dibenzothiophene (DBT), dibenzofuran (DBF), fluorene (FLU), and carbazole (CBZ). In part, these compounds were chosen because they form a coherent family of poorly flexible structural analogues that vary only by essentially isosteric substitutions at a single site. As a result, their behaviors can be compared without needing to consider major differences in shape and conformation. Moreover, each of the compounds has been crystallized in multiple previous studies and has been reported to exist in only one polymorphic form. DBF,^31−34^ FLU,^35−37^ and CBZ^38−45^ all crystallize isostructurally in the orthorhombic space group **Pnma**, whereas DBT crystallizes in the monoclinic space group **P**2_1_/*n*.^38,46−48^ As a result, the set of compounds makes it possible to examine mixed crystallization in two distinct situations, both when the components crystallize isostructurally and when they are not known to do so, despite extensive screening.
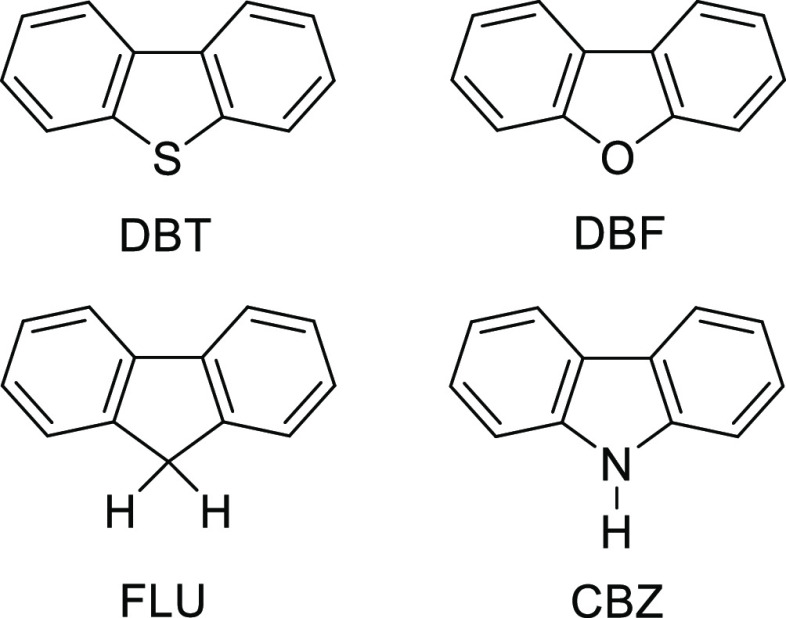


Further motivation for selecting the compounds was provided by a report that DBT, DBF, and CBZ exhibit long-lived solid-state phosphorescence,^49^ a useful phenomenon that is rare in molecular materials. Recently, the unusual emissive behavior of crystalline samples of DBT, DBF, CBZ, and related compounds has been attributed to inadvertent contamination by structurally analogous dopants.^38^ These observations show that low levels of structurally related impurities resulting from mixed crystallization can have major effects on the properties of ordered solids. Such impurities cannot necessarily be eliminated by repeated crystallizations and are best avoided by choosing routes of synthesis that do not produce them.

For these various reasons, the ability of the crystal lattices of DBT, DBF, FLU, CBZ, and their analogues to exclude or include related species is a topic of broad interest. We have prepared mixed crystals of these compounds and analyzed the series by single-crystal X-ray diffraction, thermal methods of characterization, computational modeling, and other techniques. Although our studies focus on the behavior of a specific set of compounds, they have allowed us to draw conclusions of general value and to begin to clarify the rules governing mixed crystallizations of all types.

## Results and Discussion

### Characterization of Crystals of Pure DBT, DBF, FLU, and CBZ by Single-Crystal X-ray Diffraction

**Figure 1:**
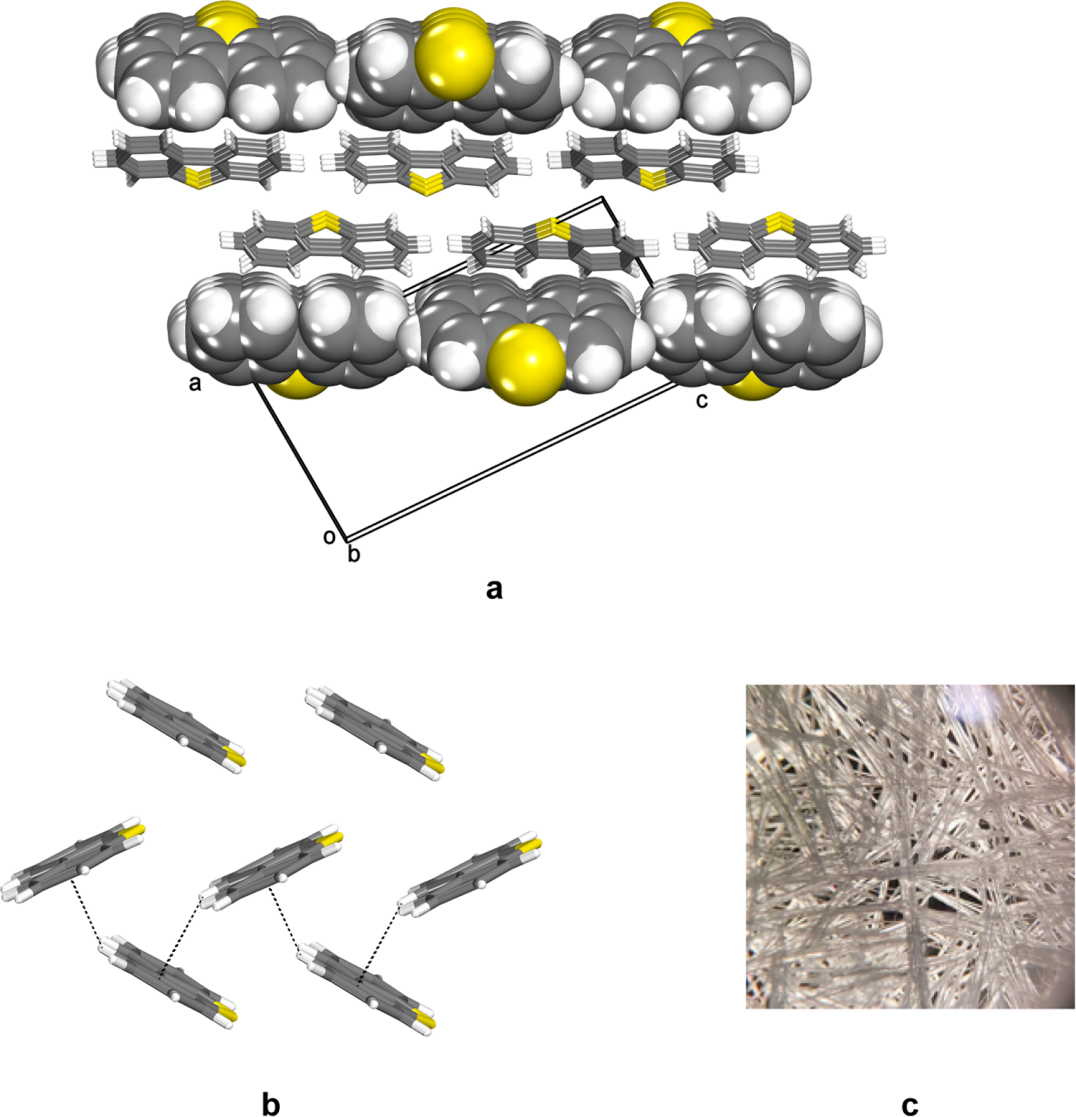
(a) Representation of the structure of monoclinic *P*2_1_/*n* crystals of DBT,^47^ as viewed along the *b*-axis. (b) View of molecules linked along the *b*-axis by C–H···π interactions (broken lines). (c) Optical micrograph showing an area of approximately 1 × 1 cm^2^ containing needles formed by DBT. In the structural images, selected molecules are shown in a space-filling representation, and atoms of carbon appear in gray, hydrogen in white, and sulfur in yellow.

**Figure 2:**
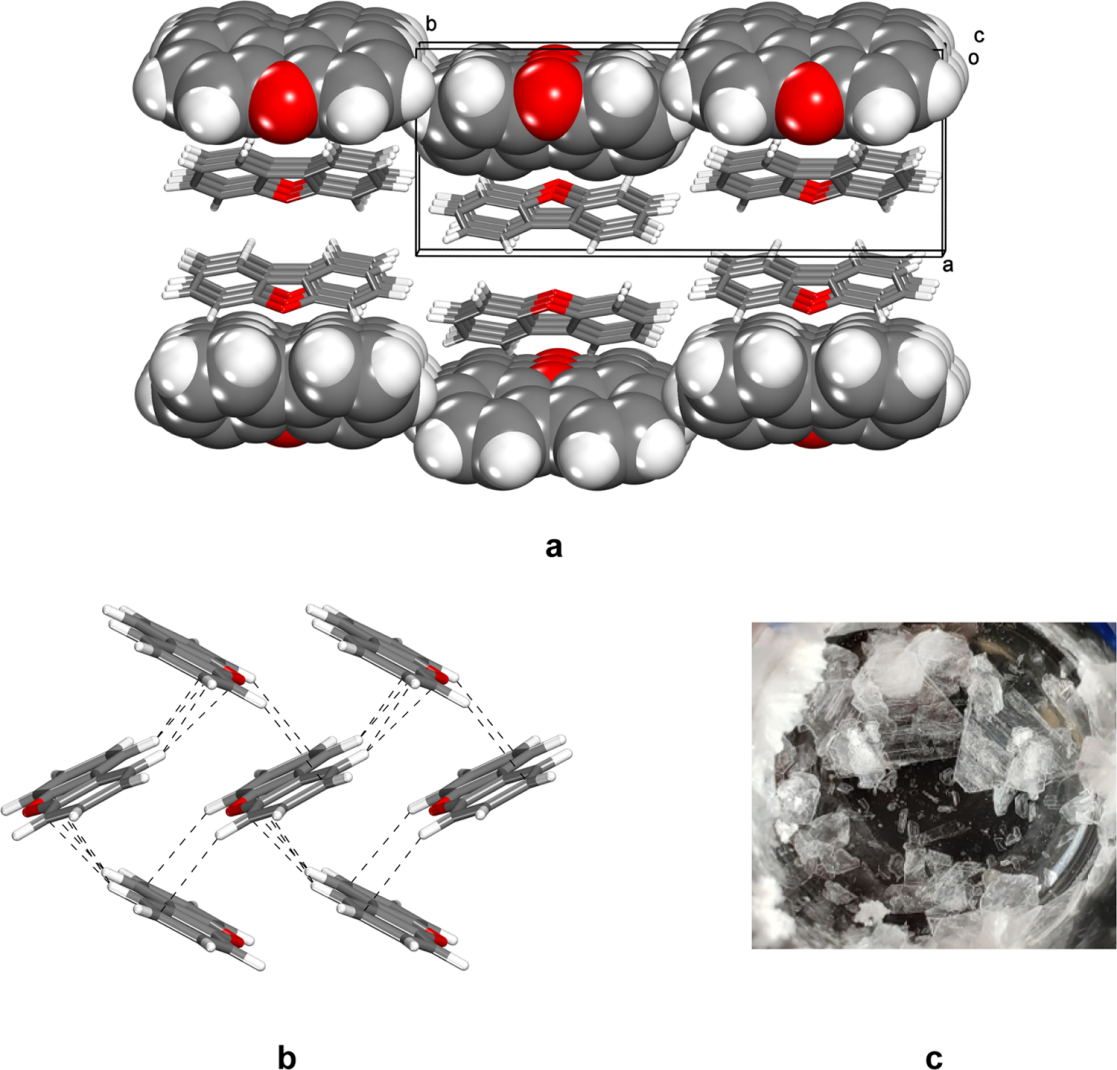
(a) Representation of the structure of orthorhombic *Pnma* crystals of DBF,^32^ as viewed along the *c*-axis. (b) Molecules linked in the *ac*-plane by C–H···π interactions (broken lines). (c) Optical micrograph showing an area of approximately 1 × 1 cm^2^ containing thin plates formed by DBF. In the structural images, selected molecules are shown in a space-filling representation, and atoms of carbon appear in gray, hydrogen in white, and oxygen in red.

Data related to the structures of crystals of pure DBT, DBF, FLU, and CBZ are summarized in Table 1, and representative views of the monoclinic **P**2_1_/*n* structure of DBT and the orthorhombic **Pnma** structure of DBF are provided in Figures 1 and 2 for comparison. These data confirm the isostructurality of DBF, FLU, and CBZ, as well as the existence of marked differences between the **P**2_1_/*n* and **Pnma** structures. In particular, the data provide a reminder that compounds differing only by swapping atoms of oxygen and sulfur do not necessarily prefer to crystallize in the same way.^28−30,50−52^Table 2 provides quantitative evaluations of the structural similarity and dissimilarity of all six possible pairs of compounds, as assessed by determining the unit-cell similarity index Π^53^ and by using the Crystal Structure Similarity tool in the program Mercury to analyze overlays in the packing of 30-molecule clusters and to measure root-mean-square deviations (RMSD_30_) of atomic positions in the overlays. As expected, the values of Π and RMSD_30_ are significantly lower for the isostructural pairs.

**Table 1:** Unit-Cell Parameters and Other Crystallographic Data for DBT, DBF, FLU, and CBZ

compound	DBT^47^	DBF^32^	FLU^35^	CBZ^40^	DBT
CSD refcode^a^	DBZTHP01	DBZFUR02	FLUREN02	CRBZOL11	
description	colorless needles	colorless plates	colorless plates	colorless plates	colorless plates
crystal syst	monoclinic	orthorhombic	orthorhombic	orthorhombic	orthorhombic
space group	**P**2_1_/*n*	**Pnma**	**Pnma**	**Pnma**	**Pnma**
*a* (Å)	8.551(5)	7.5154(8)	8.365(3)	7.6371(2)	8.0529(8)
*b* (Å)	5.956(5)	19.098(2)	18.745(4)	19.0042(6)	18.8619(17)
*c* (Å)	16.994(5)	5.7739(6)	5.654(2)	5.6776(1)	5.8033(4)
α (deg)	90	90	90	90	90
β (deg)	94.393(5)	90	90	90	90
γ (deg)	90	90	90	90	90
*V* (Å^3^)	863.0(9)	828.7(2)	886.4(5)	824.03(4)	881.48(13)
*Z*	4	4	4	4	4
*Z*′	1	0.5	0.5	0.5	0.5
ρ_calc_ (g·cm^–3^)	1.418	1.348	1.245	1.348	1.388
*T* (K)	100	169	159	100	100
*R*_1_, *I* > 2*σ*(*I*)	0.0346	0.039	0.043	0.033	0.0427
w**R**_2_, *I* > 2*σ*(*I*)	0.0882	0.040	0.045	0.093	0.1074
GoF	1.002			1.08	1.070
packing coefficient^b^	0.730	0.717	0.707	0.727	0.705

a Cambridge Structural Database (CSD).

b Kitaigorodsky packing coefficient as determined using PLATON.^54^

**Table 2:** Quantitative Evaluations of the Similarity and Dissimilarity of Reported Structures of DBT, DBF, FLU, and CBZ

pair	CSD refcodes	Π^a^	common molecules (in clusters of 30)^b^	RMSD_30_ (Å)^b^
DBT/DBF	DBZTHP01/DBZFUR02	0.0296	8	2.363
DBT/FLU	DBZTHP01/FLUREN02	0.0416	8	2.541
DBT/CBZ	DBZTHP01/CRBZOL11	0.0274	8	2.218
DBF/FLU	DBZFUR02/FLUREN02	0.0116	30	0.476
DBF/CBZ	DBZFUR02/CRBZOL11	0.0021	30	0.123
FLU/CBZ	FLUREN02/CRBZOL11	0.0138	30	0.387

a Unit-cell similarity index.^53^

b Assessed using the Crystal Structure Similarity tool in the program Mercury.

No intermolecular contacts in any of the reported structures of DBT, DBF, FLU, and CBZ are much shorter than the sum of the van der Waals radii of the atoms involved, and typical herringbone packing is observed (Figures 1 and 2). The monoclinic **P**2_1_/*n* structure of crystals of DBT can be considered to be built from chains of molecules linked along the *b*-axis by C–H···π interactions with H···C distances (*d*_H···C_) of 2.820 and 2.888 Å (Figure 1b). The orthorhombic **Pnma** structure of crystals of DBF is formed from sheets of molecules joined in the *ac*-plane by C–H···π interactions with values of *d*_H···C_ in the range 2.827–2.999 Å. The efficiency of packing in both structures is normal, as assessed using PLATON to measure Kitaigorodsky coefficients (Table 1).^54^ Crystallization of DBT from MeOH typically produced colorless needles (Figure 1c), whereas DBF, FLU, and CBZ crystallized under similar conditions as thin colorless plates (Figure 2c). Indexation of the crystals revealed that growth is fastest along the *b*-axis in the case of DBT and in the *ac*-plane in the cases of DBF, FLU, and CBZ. In all cases, growth is fastest in directions aligned with the formation of primary C–H···π interactions.

**Figure 3:**
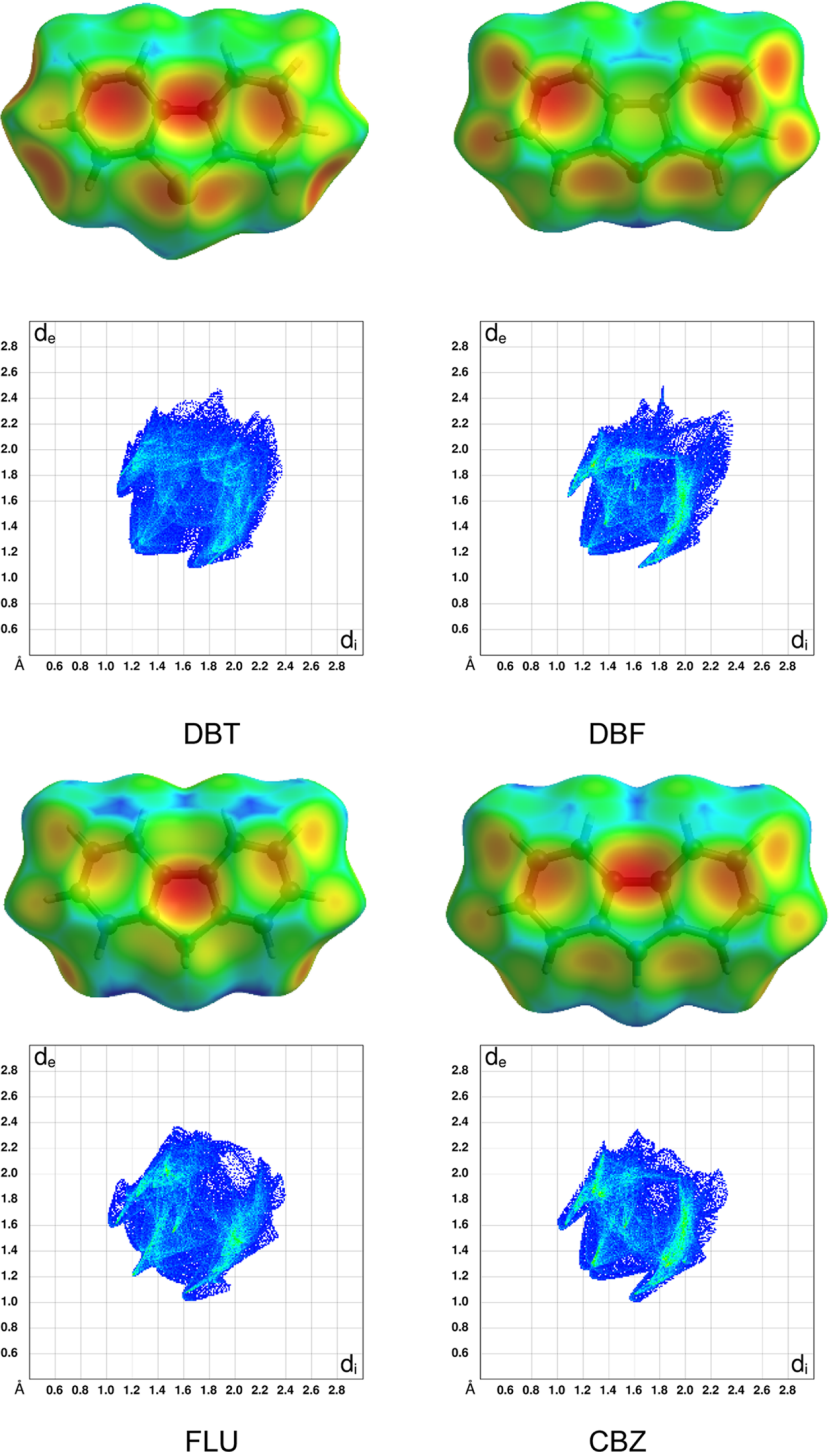
Hirshfeld surfaces (top images) and the corresponding two-dimensional fingerprint plots (bottom images) for molecules in monoclinic *P*2_1_/*n* crystals of DBT and orthorhombic *Pnma* crystals of DBF, FLU, and CBZ. The Hirshfeld surfaces are colored according to the local value of *d*_e_ (distance from the surface to the nearest atomic nucleus in another molecule), and the colors range from cool (blue) to hot (red) as *d*_e_ decreases. The fingerprint plots show the frequency of finding points on the surface with particular values of *d*_e_ and *d*_i_ (distances to the nearest external and internal atomic nuclei). The colors at each point range from cool (blue) to hot (red) as the frequency increases.

To compare interactions in the monoclinic **P**2_1_/*n* crystals of DBT with those in the orthorhombic **Pnma** crystals of DBF, FLU, and CBZ, we constructed Hirshfeld surfaces and related two-dimensional fingerprint plots (Figure 3).^55,56^ The Hirshfeld surface of a molecule in a crystal defines the origin of local electron density, typically by showing where the density derived from atoms in the molecule equals the density contributed by all other atoms in the structure. Colors on the surface can be varied according to parameters related to close intermolecular contacts, such as the distance from the surface to the nearest atomic nucleus in another molecule. Related fingerprint plots represent the relative number of points on Hirshfeld surfaces where distances to the nearest external atomic nucleus (*d*_e_) and to the nearest internal atomic nucleus (*d*_i_) have specific values. As the frequency of finding a particular coordinate (*d*_e_, *d*_i_) rises, the color at that point on the fingerprint plot can be varied. The surfaces and plots in Figure 3 show that the four compounds engage in similar types of interactions in the crystalline state, even though the molecular arrangement in crystals of DBT is different from the one favored by DBF, FLU, and CBZ. The fingerprint plots confirm the special importance of C-H···π interactions in the **Pnma** structures, as revealed by the relatively high frequency of points near (*d*_i_, *d*_e_) ≈ (1.9, 1.3) or (*d*_i_, *d*_e_) ≈ (1.3, 1.9).

### Crystal Structure Prediction (CSP)

**Figure 4:**
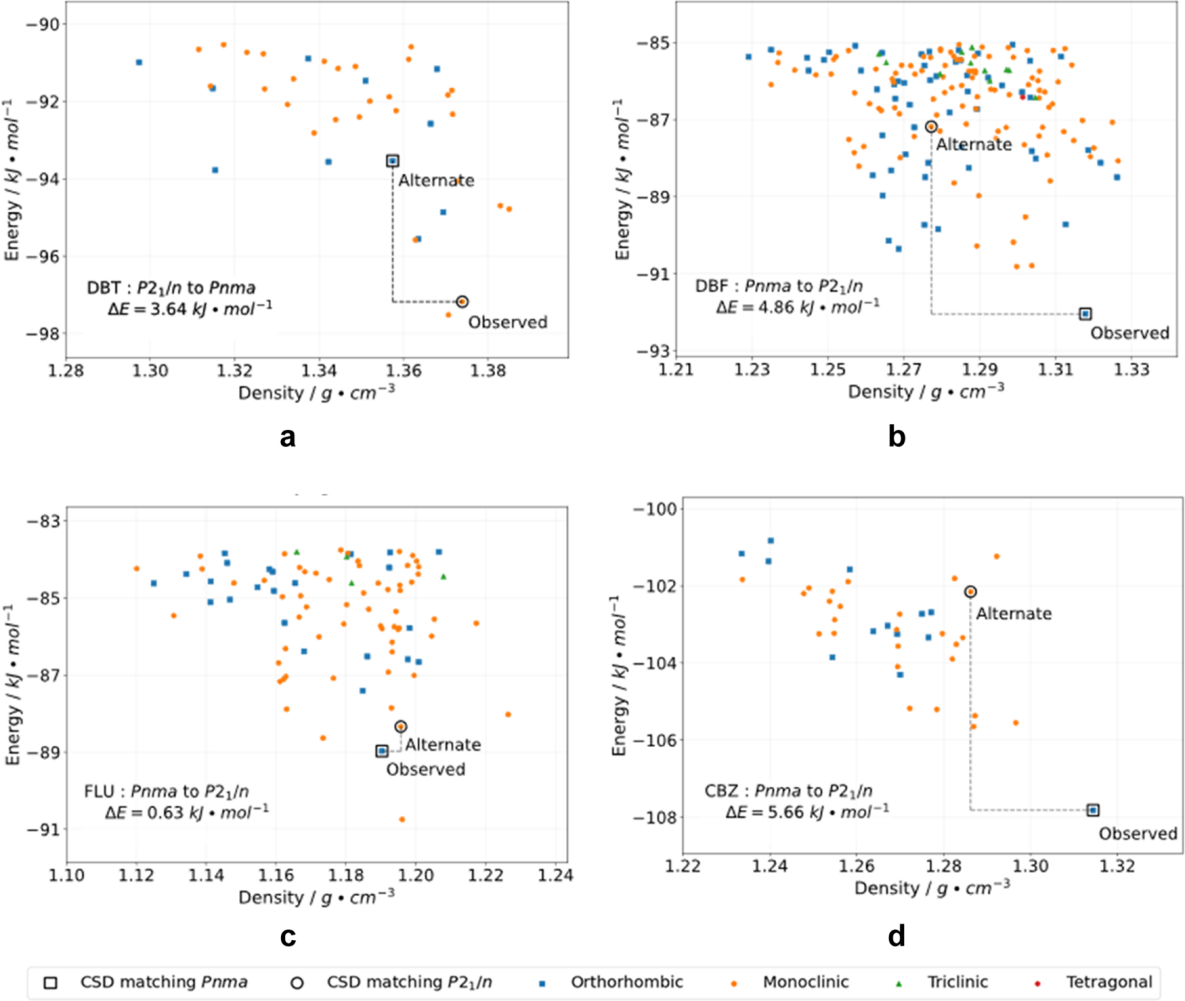
Plots showing energies and densities in the low-energy regions of the predicted polymorphic landscapes of (a) DBT, (b) DBF, (c) FLU, and (d) CBZ. Each plotted point represents a predicted crystal structure, and the color and shape of the marker identify the crystal system according to the legend provided below the plots. On each plot, two points of special interest are enclosed in black circles or squares (*P*2_1_/*n* or *Pnma* structures, respectively) and labeled as “observed” or “alternate.” These structures are highlighted because they match experimentally determined forms (“observed”) or because they are unreported but isostructural with respect to the known crystal structure of one or more of the other three compounds (“alternate”).

The crystal structure landscapes of the four compounds were mapped by CSP using quasi-random exploration of the energy surface,^57^ as defined by an empirically parametrized force field and atom multipole electrostatics. The CSP calculations are described in detail in the Supporting Information, and the results are summarized in Figure 4 and Table 3.

**Table 3:** Predicted Crystallographic Parameters for CSP-Generated Polymorphs that Match Experimental Data from the CSD, with Percent Deviations in Parentheses

compound	DBT	DBF	FLU	CBZ
CSD refcode	DBZTHP01	DBZFUR02	FLUREN02	CRBZOL11
CSP match ID	opt_DBZTHP-QR-14-14701-3	opt_DBZFUR-QR-19-11479-3	opt_flu-QR-14-3324-3	opt_cbz-QR-14-7423-3
crystal syst	monoclinic	orthorhombic	orthorhombic	orthorhombic
space group	**P**2_1_/*n*	**Pnma**	**Pnma**	**Pnma**
*a* (Å)	8.779 (+2.7%)	7.966 (+6.0%)	8.914 (+6.6%)	8.042 (+5.3%)
*b* (Å)	5.796 (−2.7%)	19.056 (−0.2%)	18.886 (+0.8%)	19.159 (+0.8%)
*c* (Å)	17.540 (+3.2%)	5.584 (−3.3%)	5.510 (−2.3%)	5.484 (−3.4%)
α (deg)	90	90	90	90
β (deg)	93.59 (−0.9%)	90	90	90
γ (deg)	90	90	90	90
*V* (Å^3^)	890.794 (+3.2%)	847.723 (+2.3%)	927.509 (+4.6%)	844.936 (+2.5%)
ρ_calc_ (g·cm^–3^)	1.3739	1.3178	1.1903	1.3144
RMSD_30_ (Å)^a^	0.276	0.265	0.319	0.263

a RMSD in atomic positions in 30-molecule clusters taken from predicted and reported crystal structures, as calculated using the COMPACK algorithm.^58^

Although all four compounds have similar molecular structures, the number of predicted low-energy forms varies markedly. For example, the landscape of DBT is relatively sparse (Figure 4a), whereas that of DBF has many predicted structures (Figure 4b). These results show that small changes in molecular structure can not only alter crystal packing but can also have a large global impact on the features and complexity of the crystal energy landscape. CSP was able to reproduce the known crystal structure of each compound with deviations in unit-cell parameters less than 7% and with values of RMSD_30_ less than 0.32 Å (Table 3). As shown in Figure 4, the observed crystal structures are predicted to be either the global minimum-energy forms (DBF and CBZ) or the second-lowest structures (DBT and FLU, located 0.34 and 1.78 kJ·mol^–1^ above the predicted global energy minima, respectively).

The results of the CSP studies validate the force field used for modeling, and they also provide a measure of the difference in energy between the two types of packing observed (**Pnma** and **P**2_1_/*n*). For DBT, the calculations predict a polymorph isostructural to the known **Pnma** structures of DBF, FLU, and CBZ at a calculated energy of 3.64 kJ·mol^–1^ above that of the observed **P**2_1_/*n* structure. The difference in energy is within the range normally observed among polymorphs,^59^ suggesting that the **Pnma** structure of DBT might be accessible. In addition, the predicted energy landscapes of DBF, FLU, and CBZ include **P**2_1_/*n* polymorphs that are isostructural to the known form of DBT and have energies that lie at 4.86, 0.63, and 13.18 kJ·mol^–1^, respectively, above those of their known **Pnma** forms.

### Formation of Mixed Crystals of DBT and DBF

A 1938 report predating structural analyses of DBT and DBF concluded that the two compounds form a single mixed crystalline phase in all proportions.^60^ However, a more recent calorimetric study indicated that the nonisostructural pairs DBT/DBF and DBT/FLU form mixed crystals with narrower ranges of compositions, whereas isostructural DBF and FLU are miscible in the solid state in all ratios.^61^ The findings of the calorimetric study support the general conclusions of Kitaigorodsky and co-workers about the mixed crystallization of isostructural pairs. However, the calorimetric study also reveals surprises that underscore how poorly the phenomenon of mixed crystallization is understood in other cases. For example, the published solid–liquid phase diagram of DBT/DBF suggests that mixed crystals with the orthorhombic **Pnma** structure of DBF will be formed at any molar fraction of DBF (χ_DBF_) in the approximate range 1.0 > χ_DBF_ > 0.2. This is noteworthy for two reasons: (1) The components are not known to crystallize isostructurally, yet they can coexist in a single crystalline phase in ratios varying continuously over a very wide range; and (2) the **Pnma** structure is retained throughout, even though molecules of DBF are replaced by a larger analogue that prefers an alternative packing in pure form.

To test the implications of the phase diagram, we crystallized DBT and DBF from solutions in MeOH containing ratios of the two components varying in the approximate range 1.0 > χ_DBF_ > 0.2, and we examined the resulting mixed crystals by multiple techniques. Table 4 summarizes data obtained by single-crystal X-ray diffraction. All crystals obtained in these experiments were thin plates, as observed in **Pnma** crystals of pure DBF. Compositions were determined by carefully refining the relative occupancy of atoms of oxygen and sulfur while using similarity constraints on the atomic displacement parameters. The DBT/DBF ratios measured by X-ray diffraction in individual crystals matched those present in the initial solutions within approximately 10%. DBT was also observed to form binary mixed crystals with FLU and CBZ. Extensive studies of the structure and composition of these additional mixed crystals were not carried out, but results similar to those in Table 4 were obtained. For example, crystallization of a 1:1 mixture of DBT and FLU gave mixed **Pnma** crystals with a representative DBT/FLU ratio of 0.22:0.78, and crystallization of a 1:1 mixture of DBT and CBZ gave mixed **Pnma** crystals with a representative DBT/CBZ ratio of 0.62:0.38. Deviations in composition from the nominal ratio of components in solution may reflect differences in solubility, as well as selective incorporation during the growth of crystals. Ternary mixed crystals containing DBT and two components selected from among its isostructural analogues DBF, FLU, and CBZ could also be grown. Further descriptions of mixed crystals other than those containing only DBT and DBF are provided in the Supporting Information.

**Table 4:** Selected Crystallographic Data for Mixed Crystals of DBT and DBF

compound	DBT/DBF mixed crystals				
DBT/DBF ratio (crystal)^a^	0.23:0.77	0.46:0.54	0.59:0.41	0.73:0.27	0.79:0.21
DBT/DBF ratio (initial solution)	2:8	4:6	5:5	7:3	8:2
CSD refcode	2195728	2195719	2195725	2195727	2195723
crystal syst	orthorhombic	orthorhombic	orthorhombic	orthorhombic	orthorhombic
space group	**Pnma**	**Pnma**	**Pnma**	**Pnma**	**Pnma**
*a* (Å)	7.6368(4)	7.7875(3)	7.8867(5)	7.9686(3)	8.0114(7)
*b* (Å)	18.9566(13)	18.9102(8)	18.8866(12)	18.8543(6)	18.8298(16)
*c* (Å)	5.7980(4)	5.8053(3)	5.8042(4)	5.8060(2)	5.8063(5)
*α* (deg)	90	90	90	90	90
*β* (deg)	90	90	90	90	90
*γ* (deg)	90	90	90	90	90
*V* (Å^3^)	839.36(9)	854.91(7)	864.55(10)	872.31(5)	875.90(13)
*T* (K)	100	100	100	100	100

a As determined by single-crystal X-ray diffraction.

In selected cases, several mixed crystals of DBT and DBF were chosen at random from the same batch, examined by X-ray diffraction, and shown to have similar compositions. The compounds proved to be too volatile to allow the compositions of individual mixed crystals to be determined routinely by energy-dispersive X-ray spectroscopy. However, we found that characteristic differences in the Raman spectra of DBT and DBF, particularly in the region 200–1100 cm^–1^,^62−64^ can be used to determine the local ratio of components in single mixed crystals. Particularly useful bands are those attributed to in-plane C–C–C bending near 701 cm^–1^ (υ_701_) for DBT (in pure **P**2_1_/*n* crystals) and near 730 cm^–1^ (υ_730_) for DBF (in pure **Pnma** crystals). The relative intensity of these bands, as measured by Raman microspectroscopy, confirmed the accuracy of compositions determined by X-ray diffraction. Different crystals in each batch, as well as different positions in individual crystals, could be shown to have the same DBT/DBF ratios within about 10–30% (Table 5).

**Table 5:** Composition of Individual Mixed Crystals of DBT and DBF as Analyzed by Raman Microscopy and ^1^H NMR Spectroscopy

entry	initial DBT/DBF ratio in solution	local DBT/DBF ratios at 5 random positions (Raman)^a^					average ratio	overall DBT/DBF ratio (^1^H NMR)^b^
1	0.43	0.53	0.69	0.70	0.61	0.50	0.61	0.60
2	0.43	0.55	0.57	0.74	0.74	0.64	0.65	0.63
3	1.5	2.0	1.9	2.3	3.2	2.6	2.4	2.1
4	1.5	1.6	2.0	1.4	1.5	1.6	1.6	1.6

a As assessed by Raman microscopy, using the relative intensities of bands at υ_701_ (DBT) and υ_730_ (DBF).

b As measured by dissolving the individual crystals analyzed by Raman microscopy and integrating peaks in the ^1^H NMR spectra.

**Figure 5:**
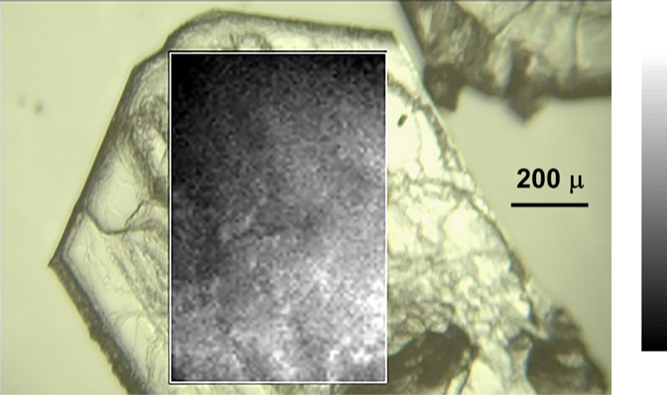
Optical micrograph showing a mixed *Pnma* crystal grown from a solution in MeOH containing an initial DBT/DBF ratio of 2:3, with an overlaid compositional map of a rectangular part of the crystal obtained by Raman microscopy. Local composition was determined by measuring the relative intensities of characteristic Raman bands (υ_701_ for DBT and υ_730_ for DBF). The scale ranges from black to white as the local value of χ_DBT_ increases from 0.33 to 0.50.

Further evidence of homogeneity was provided by mapping the surfaces of single mixed **Pnma** crystals of DBT and DBF, using Raman microscopy to measure the local ratio of the intensities of the υ_701_ and υ_730_ bands (Figure 5). In assessing the map, it is important to note that the nominal ratio of components is χ_DBT_ = 0.40, and the entire black-to-white scale corresponds to variation only within the range 0.33 ≤ χ_DBT_ ≤ 0.5. The small variation suggests that stopping crystallization at various points would not alter the stoichiometry significantly. Further confirmation of composition was provided by taking crystals examined by Raman microscopy, dissolving them individually in CDCl_3_, and analyzing the solutions by ^1^H NMR spectroscopy (Table 5). Together, our results reveal that mixed **Pnma** crystals of DBT and DBF spanning a wide range of compositions grow with little discrimination of the components, despite the lack of established isostructurality. In contrast, **P**2_1_/*n* single crystals derived from 9:1 mixtures of DBT:DBF in MeOH did not contain amounts of DBF that could be measured using X-ray diffraction, Raman microscopy, or ^1^H NMR spectroscopy. This suggests that the growth of **P**2_1_/*n* crystals of DBT is more selective and can exclude molecules of DBF effectively, even though they are smaller.

### Computational Modeling of Mixed Crystals

The behavior of DBT, DBF, and their analogues highlights the complexity of mixed crystallization and the underdeveloped potential of the phenomenon to produce new materials with tunable properties. The compounds confirm the feasibility of making mixed crystals with compositions and properties that change continuously over very wide ranges, even when the components do not favor isostructural crystallization. Such behavior is not unprecedented, but the governing principles are mysterious. To develop a deeper understanding of the phenomenon and to learn how to predict when it can occur, we used computational methods to estimate the energetic cost of substituting molecules of DBF in its normal **Pnma** structure with molecules of DBT across the full compositional range, from pure DBF to pure DBT. Similarly, we evaluated the corresponding cost of replacing molecules of DBT in its preferred **P**2_1_/*n* structure with molecules of DBF. Calculations of this type have rarely been used in previous studies of mixed crystallization, but they have significant potential for assessing the feasibility of the phenomenon and the range of accessible compositions.^65−70^

To create mixed-crystal models that allow sufficiently small increments in composition and that minimize artifacts, such as those arising from the effect of periodic boundary conditions on the random mixing of components, 32-molecule supercells based on the *Z* = 4 crystal structures of DBT and DBF were built. For each composition, randomly chosen molecules in the supercells of the host compound were replaced by isostructural imposters, with their atomic positions overlaid on those of the host as closely as possible. To probe the possible effect of placing imposters in alternative sites in the supercells, 40 distinct configurations were assayed for each composition, differing in which randomly selected molecules were replaced. Our study has focused on a small set of structurally related compounds that have been reported to crystallize in only two space groups, and one of them (**P**2_1_/*n*) is a subgroup of the other (**Pnma**); nevertheless, the computational methodology we use to assess the feasibility of mixed crystallization is not limited to the study of compounds crystallizing in specific space groups. In the cases of DBT, DBF, and their analogues, replacing molecules in supercells was straightforward because the molecular shapes are closely similar. In applying the method to sets of molecules that are more dissimilar, we plan to add a step after molecular replacement, in which clashes between molecules are detected and relieved by approaches used in our CSP methods.^57^ In such ways, the approach we have followed promises to be generally useful.

**Figure 6:**
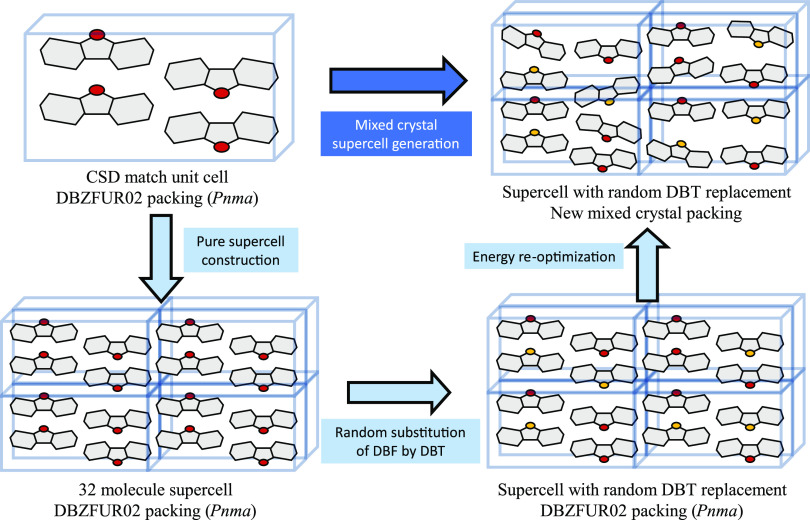
Overview of the method for generating and optimizing mixed-crystal supercells, as illustrated by partially replacing DBF with DBT in the normal *Pnma* packing of DBF.

In total, 2640 DBT/DBF mixed-crystal supercells were constructed and energy-optimized, based on two packing arrangements (the normal **P**2_1_/*n* structure of DBT and the **Pnma** structure of DBF), 40 sets of randomly swapped molecules, and 33 equally spaced DBF/DBT ratios (1:0, 0.97:0.03, 0.94:0.06, 0.91:0.09, ··· 0:1). Figure 6 summarizes the method employed, and a more detailed description is provided in the Supporting Information.

**Figure 7:**
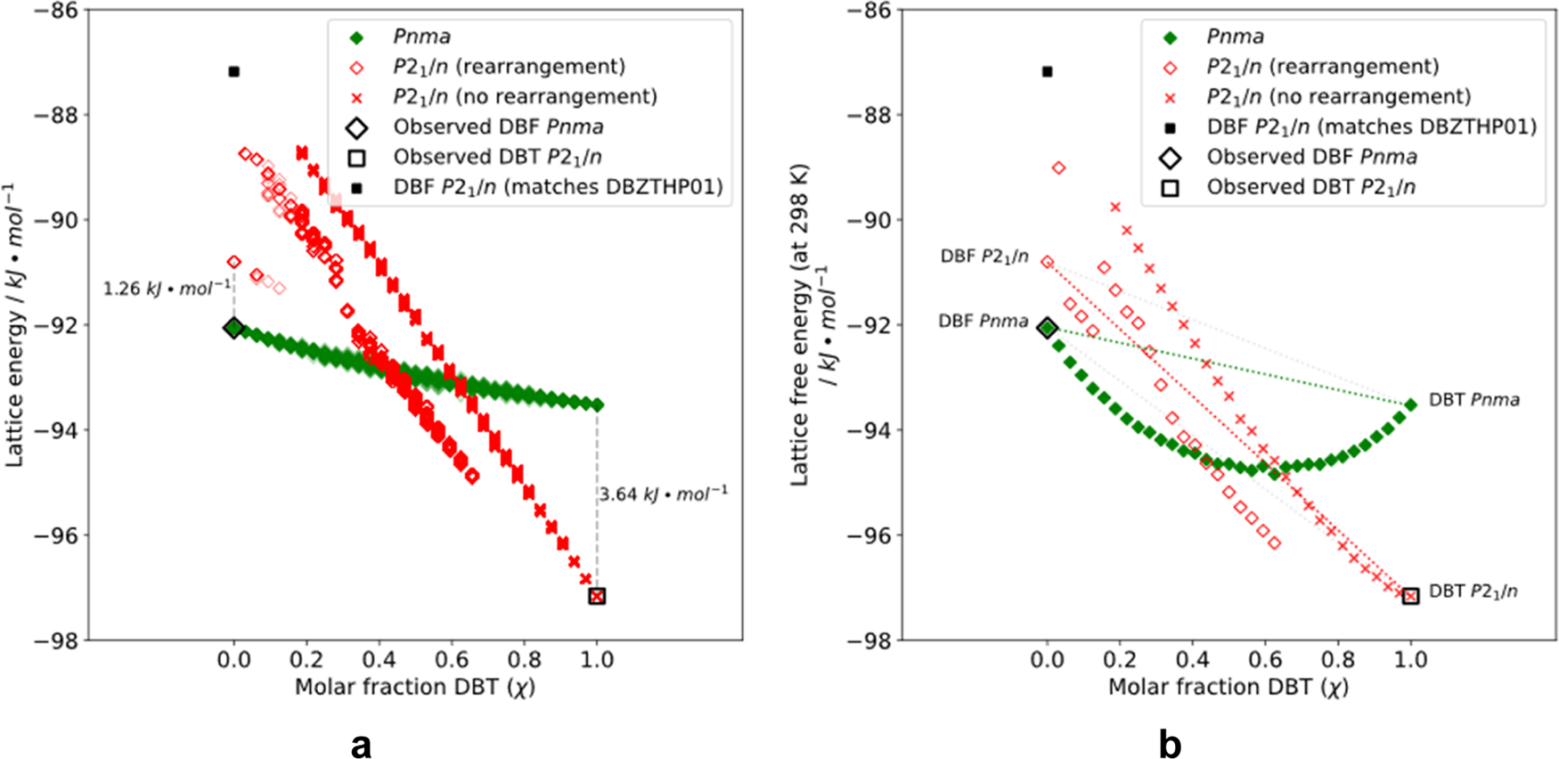
(a) Plots showing the relationship between χ_DBT_ and the lattice energies of mixed crystals of DBF and DBT, as calculated for structures in which DBF and DBT have been swapped in their normal *Pnma* and *P*2_1_/*n* packing arrangements (green and red data points, respectively). Structures marked as “observed” (open black diamonds and squares) correspond to predicted structures that match the experimentally observed DBF and DBT structures taken from the CSD and used as the initial packing arrangements to construct mixed-crystal models. The energy of the predicted DBF structure that is isostructural to *P*2_1_/*n* crystals of DBT is shown as a solid black square. Red crosses correspond to *P*2_1_/*n* mixed-crystal configurations that maintain the same molecular packing as in *P*2_1_/*n* crystals of pure DBT after minimization of the lattice energy. Open red diamonds denote *P*2_1_/*n* mixed-crystal configurations that undergo structural rearrangement during energy minimization. (b) Plots of the free energy of simulated mixed crystals of DBT and DBF as a function of χ_DBT_ at 298 K, with dashed lines corresponding to the composition-weighted energy of the pure unmixed components.

The energies of mixed crystals of DBT and DBF calculated by this method are plotted as a function of χ_DBT_ in Figure 7. The lattice energy of the **Pnma** phase (green dots, Figure 7a) was found to change smoothly with composition, and the 40 configurations examined for each composition have essentially the same energy. When all molecules of DBF were replaced by DBT in the **Pnma** packing, optimization led to the isostructural form identified on the CSP landscape of DBT (Figure 4a), which is calculated to be 3.64 kJ·mol^–1^ less stable than the known **P**2_1_/*n* form (CSD reference code DBZTHP01). The energy of the **Pnma** mixed crystals was calculated to decrease by about 1.5 kJ·mol^–1^ from pure DBF to pure DBT, due to the larger size of DBT and more significant dispersion interactions involving atoms of sulfur. The results are in agreement with the observed formation of **Pnma** mixed crystals over a wide range of compositions, as well as with the high degree of compositional uniformity seen within batches of crystals and within individual crystals (Table 5 and Figure 5).

Mixed crystals with the **P**2_1_/*n* packing favored by DBT (red data points, Figure 7a) are predicted to show markedly different behavior. In particular, the calculated energy changes much more steeply with composition, giving a slope of energy vs χ_DBT_ about 7-fold higher in **P**2_1_/*n* crystals than in the **Pnma** form. The significantly different energetic perturbation required to replace DBT by DBF may explain why **P**2_1_/*n* crystals of DBT exclude DBF. Another difference in behavior is that the energy of the **P**2_1_/*n* mixed-crystal models depends markedly on how imposter molecules of DBF are configured within the host crystal structure of DBT. As the DBT content decreases, the mixed-crystal configurations are predicted to give rise to two distinct energy pathways at molar fractions χ_DBT_ below 0.62. At this point, certain configurations (open red diamonds in Figure 7) undergo a structural rearrangement upon energy minimization, leading to an alternative mode of packing that is approximately 1.5 kJ·mol^–1^ lower in energy. Configurations shown as red crosses in Figure 7 maintain the original packing of the **P**2_1_/*n* form of pure DBT. The configurations split again near χ_DBT_ = 0.2, and when all molecules of DBT are replaced by DBF in the **P**2_1_/*n* form, the resulting optimized structure is no longer the closest match on the CSP landscape of DBF (indicated as a solid black square in Figure 7). Instead, the crystal is predicted to be transformed into an alternative **P**2_1_/*n* structure of lower energy and higher density, which corresponds to the form of second-lowest energy predicted in the CSP study of DBF (1.26 kJ·mol^–1^ above the known **Pnma** structure, CSD reference code DBZFUR02). A comparison of these two **P**2_1_/*n* structures of DBF is shown in Figure S7 in the Supporting Information.

The structural transformation in the **P**2_1_/*n* mixed-crystal model was explored further using different supercells of the parent **P**2_1_/*n* structure of DBT, as shown in Figures S12–S14 in the Supporting Information. Although transformation to the denser structure was observed at high values of χ_DBF_ in all supercells, it occurred over a wider range of compositions when the supercell was expanded along the *a*-axis, but only at high values of χ_DBF_ in supercells expanded solely along *b* or *c*. These results, along with visualization of the energy-minimized mixed-crystal structures, indicate that transformations of hypothetical **P**2_1_/*n* mixed crystals are sensitive to ordering of the components along *a*, where the intermolecular interactions are dominated by edge-to-face contacts between aromatic rings.

Our approach is noteworthy because it shows how the feasibility of mixed crystallization can be assessed computationally. The calculations correctly predict that the normal **P**2_1_/*n* packing of DBT poorly tolerates the inclusion of DBF, whereas the normal **Pnma** packing of DBF readily accommodates DBT. In addition, the calculations yield valuable insights that empirical approaches cannot readily provide. For example, optimal crystal packing and energy appear to depend significantly on how DBT and DBF are arranged in the **P**2_1_/*n* structure, which may prevent the formation of uniform mixed crystals. Moreover, our work establishes computationally that binary mixed crystals are not necessarily isostructural with respect to at least one of the two components. Such anomalies, which have been observed experimentally but not investigated extensively,^71^ highlight the complex behavior of mixed crystals and may help explain how they can serve as seeds for inducing crystallization of the components in ways that have not previously been observed.^28^

Computational modeling of the type we have carried out is valuable because it can be used to predict whether two compounds will yield mixed crystals or will crystallize separately as pure phases, based on comparing the free energies of the alternative products. For the case of DBT and DBF, Figure 7b shows how the free energies of various forms depend on χ_DBT_. The free energies of mixed crystals have been estimated by including configurational entropy, as determined by *S*_config_ = −*k*_B_∑*P*_*n*_ ln *P*_*n*_, where the sum is over all possible configurations, and the distribution of probabilities is estimated from the energies of the 40 sampled configurations at each composition. The free energy of **Pnma** mixed crystals proved to be lower than the weighted sum of the free energies of the pure components in their **Pnma** structures. In contrast, **P**2_1_/*n* mixed crystals are only marginally more stable than the pure individual **P**2_1_/*n* phases over a small compositional range at high χ_DBT_, unless rearrangement to the alternative lower-energy **P**2_1_/*n* packing is allowed. Compared with the weighted sum of the free energies of pure DBT (in its normal **P**2_1_/*n* phase) and pure DBF (in its normal **Pnma** phase), the free energy of **Pnma** mixed crystals is predicted to be lower in the range 0 < χ_DBT_ < 0.52. The computational model does not predict that **Pnma** mixed crystals should also form at even higher values of χ_DBT_, as observed experimentally. This small discrepancy may be due to limitations of the force field used or to neglect of further effects, such as vibrational contributions to the entropy.

### Behavior of Mixed Crystals of DBT and DBF

**Figure 8:**
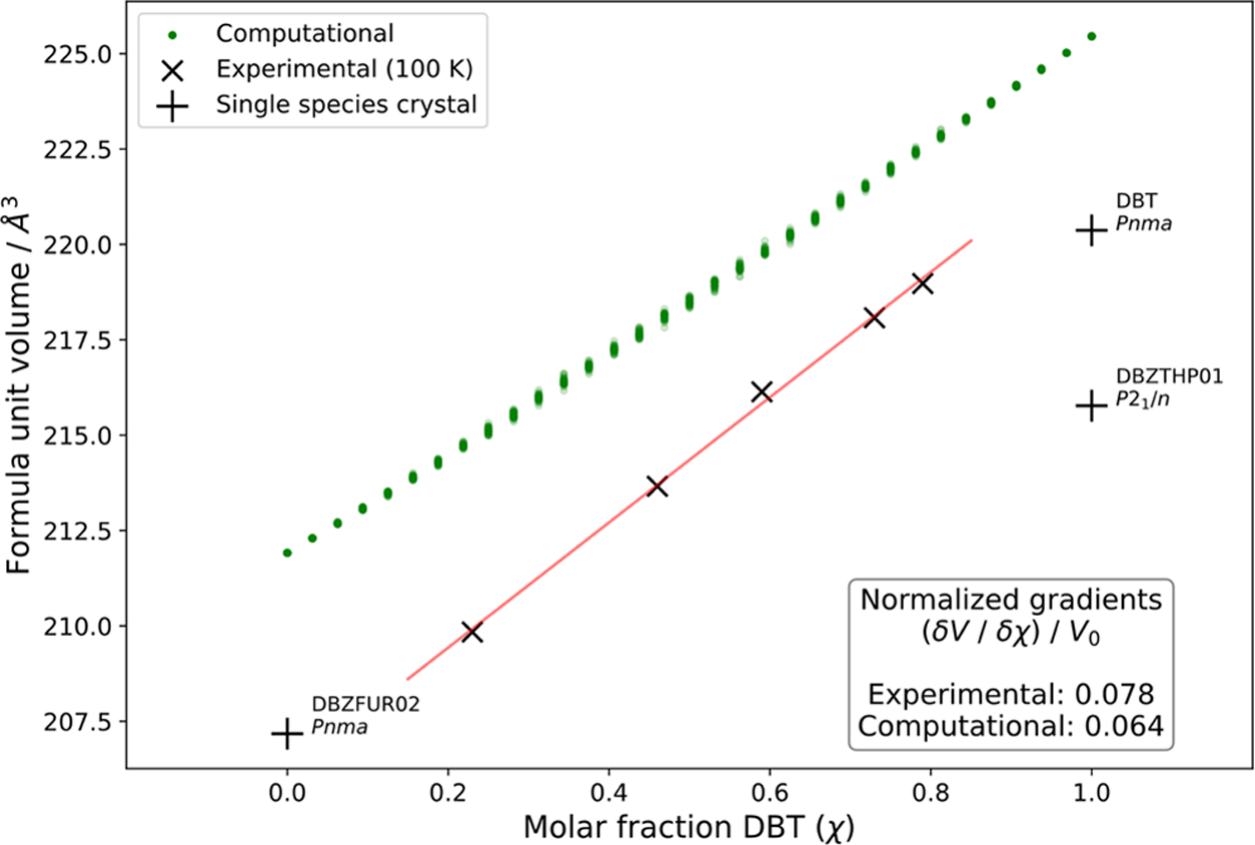
Plots of the formula unit volume of *Pnma* mixed crystals of DBT and DBF as a function of χ_DBT_. Experimental data points (×) were obtained by single-crystal X-ray diffraction at 100 K (Table 4). Computational results (green dots) exhibit a similar volume increase in simulated *Pnma* mixed crystals. The plot also includes reported values of formula unit volume (+) for *P*2_1_/*n* crystals of pure DBT at 100 K and for *Pnma* crystals of pure DBF at 169 K.^32,47^

When isostructural pairs form mixed crystals, the unit-cell parameters often vary linearly as the ratio of the components changes. This relationship, which is known as Vegard’s law,^72−74^ is not necessarily obeyed by mixed crystals of nonisostructural pairs, and few Vegard-like relationships of this type have been documented.^50^ Mixed crystals of DBT and DBF show this behavior, and a plot of the unit-cell volume as a function of χ_DBT_ is shown in Figure 8. Remarkably, an excellent linear fit is obtained, even though the components do not prefer to crystallize isostructurally, and the lattice must accommodate increasing amounts of a larger molecule. Close examination of Table 4 shows that as χ_DBT_ increases, the unit-cell parameter *c* remains essentially constant, *b* becomes slightly smaller, and *a* increases markedly. This leads to a distinctly anisotropic expansion of the unit cell, possibly because the closest O···O separations in **Pnma** crystals of DBF are much shorter along the *a*-axis (3.766 Å) than along the *b*-axis (9.976 Å) or the *c*-axis (5.774 Å). The increasing unit-cell volume of **Pnma** mixed crystals that we observe in our computational model as DBF is replaced by DBT follows a linear relationship close to that of the experimental results (Figure 8). The mixed-crystal models also reproduce the anisotropy of this expansion (Figure S10 in the Supporting Information), with most expansion occurring along *a*, very slight expansion along *c*, and nonlinear behavior of the *b* parameter, which contracts with increasing χ_DBT_ up to about 0.4, after which it becomes larger.

**Figure 9:**
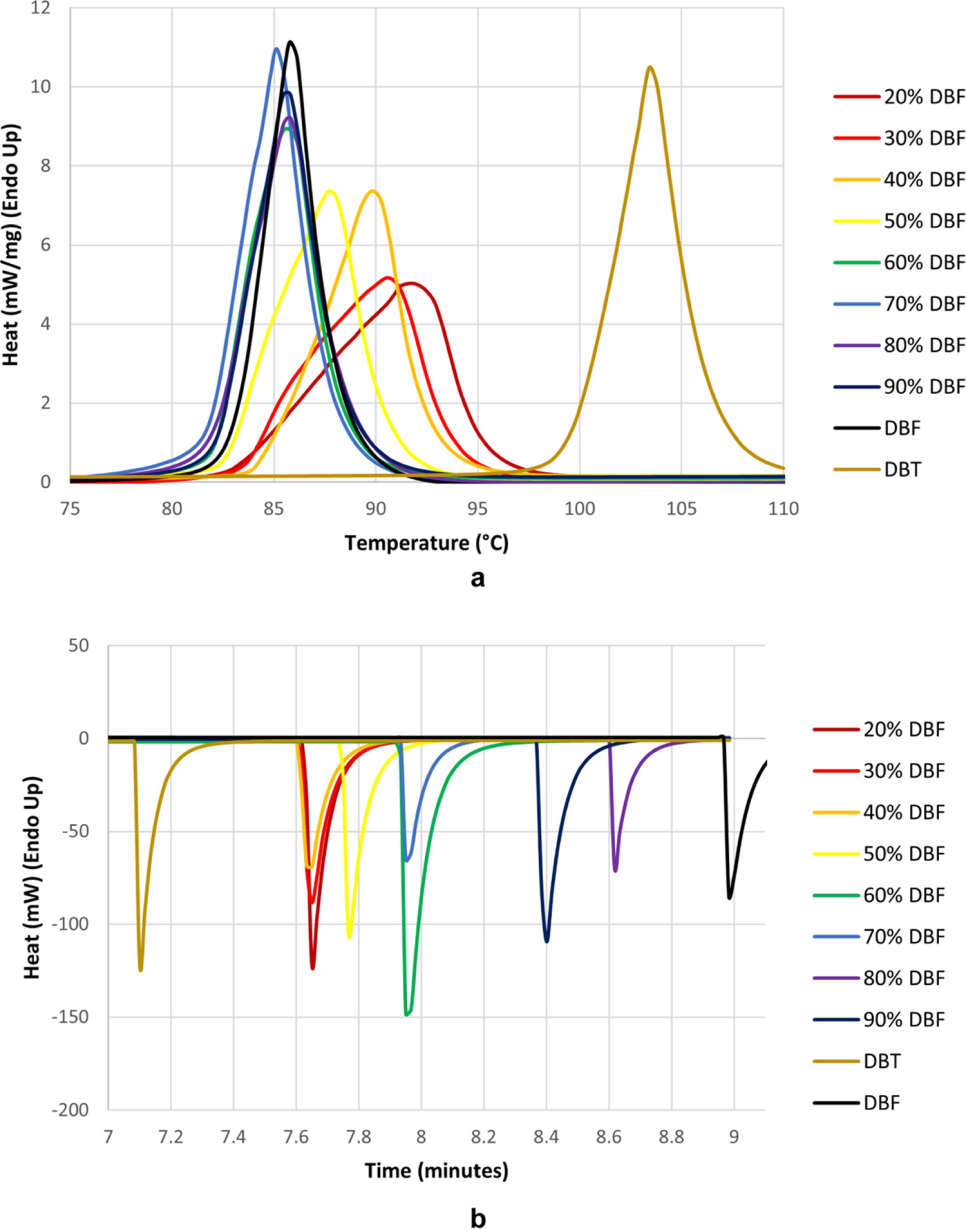
Analyses of *Pnma* mixed crystals of DBT and DBF by differential scanning calorimetry, with related data for *P*2_1_/*n* crystals of pure DBT and *Pnma* crystals of pure DBF added for comparison. (a) Melting endotherms as a function of composition. (b) Recrystallization exotherms as a function of composition. The colors of the scans identify the compositions according to the legends. In (a), heat flow is plotted as a function of temperature, which was increased at a rate of 10 °C/min. Data in (b) are shown as a function of time to avoid distortions caused by self-heating of samples during crystallization. At the start of the experiment (*t* = 0), the temperature was 30 °C. After a hold of 1 min, the temperature was raised to 110 °C at a rate of 20 °C/min, then cooled back to 30 °C at the same rate.

Analyses of **Pnma** mixed crystals of DBT and DBF by differential scanning calorimetry are shown in Figure 9, with related data for **P**2_1_/*n* crystals of pure DBT and **Pnma** crystals of pure DBF added for comparison. In mixed crystals containing mostly DBF, small amounts of DBT slightly depress the onset temperature of melting (Figure 9a), and samples with relatively large amounts of DBT show broadened endotherms at temperatures between the melting points of pure DBT and DBF. As expected, the thermal behavior of the mixed crystals is unlike that of physical mixtures of the components, which show distinct melting events. Scans obtained by cooling the melts showed sharp exotherms corresponding to recrystallization (Figure 9b). In all cases, the mixed melts crystallized at lower degrees of supercooling than required for pure DBF, suggesting that adding DBT facilitates crystallization. When various regions in recrystallized mixed melts were examined by Raman microspectroscopy, no major variations in the concentrations of the components were observed, so crystallizations from melts and solutions both occur with little discrimination of DBT and DBF.

### Mixed Crystallization, Heteroseeding, and the Discovery of New Polymorphs

The feasibility of obtaining **Pnma** mixed crystals with high values of χ_DBT_, as demonstrated both experimentally and computationally, compelled us to try to make the **Pnma** polymorph of pure DBT. As shown in Figure 4a, this potential new form appears on the predicted polymorphic landscape of DBT. However, we expected preparation and characterization to be challenging because the **Pnma** form had not been reported previously, despite extensive structural studies of DBT spanning decades. Moreover, the form is calculated to be significantly less stable (3.6 kJ·mol^–1^) than the known **P**2_1_/*n* polymorph.

Current methods for predicting polymorphic landscapes based on calculated lattice energies overestimate the number of accessible crystalline forms.^75−78^ Certain predicted low-energy polymorphs may not be isolable and characterizable, but other forms on the landscape can presumably exist as metastable species, once conditions suitable for their formation are discovered. At present, the ability to predict possible forms has outstripped the capacity to make them, and there is no generally effective way to target a potential new form on a calculated polymorphic landscape and to devise a procedure for making it selectively. A promising strategy, which can be described as templated heteroseeding, begins by matching the targeted form with a closely related known structure on the polymorphic landscape of an analogous compound.^79−83^ Crystals of the known structure can then be used as heteroseeds in attempts to induce formation of the targeted polymorph. Alternatively, mixed crystals containing the targeted compound and structural analogues can also be tested as seeds.

**Figure 10:**
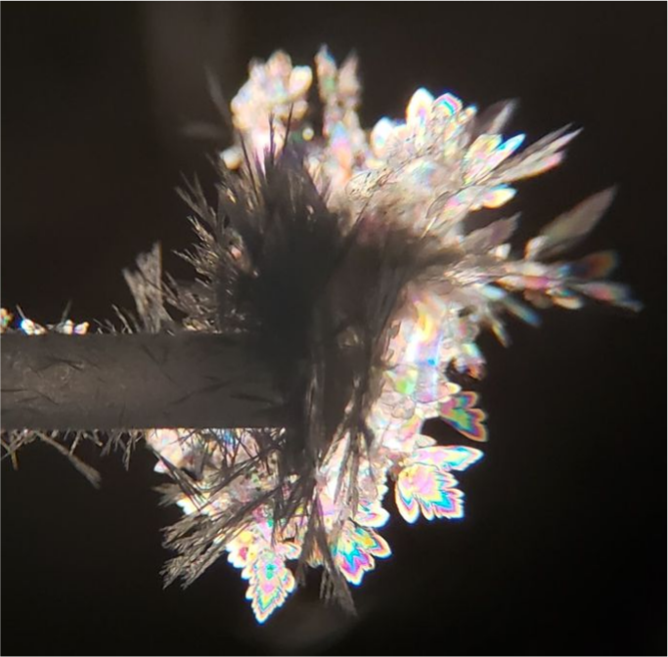
Sublimed crystals of pure DBT imaged by optical microscopy under polarized light. The thin plates correspond to the metastable *Pnma* polymorph and the needles to the previously reported *P*2_1_/*n* form.

Many attempts to induce crystallization of the putative **Pnma** polymorph of pure DBT by templation were unsuccessful. For example, when **Pnma** crystals of pure DBF or mixed **Pnma** crystals containing DBT and DBF in various ratios were used to seed the crystallization of supersaturated solutions or supercooled melts of DBT, the missing **Pnma** polymorph was not observed. Eventually, we tested the sublimation of DBT onto the surfaces of **Pnma** crystals of pure DBF, FLU, or CBZ, as well as onto the surfaces of **Pnma** mixed crystals of these compounds containing various amounts of DBT.^84^ To carry out these experiments, a seed crystal was lodged in the tip of a disposable glass pipet, and the pipet was connected to an aspirator so that vapors of DBT produced by heating the compound at atmospheric pressure could be drawn over the surface of the seed. In all cases, regardless of the composition of the **Pnma** seed, we observed the growth of very delicate thin plates on the seeds, whereas needles formed on the cool surface of the pipet itself (Figure 10).

As suggested by the distinctive morphologies of the two types of crystals, the needles proved to correspond to the known **P**2_1_/*n* form of DBT, and the thin plates were found to be crystals of the elusive **Pnma** polymorph. Sublimation in the absence of seeds yielded only the known **P**2_1_/*n* form. Structural analysis of the thin plates by single-crystal X-ray diffraction is summarized in Table 1, and quantitative comparisons of the new structure with those of the predicted form and the reported **Pnma** structures of DBF, FLU, and CBZ are provided in the Supporting Information. **Pnma** crystals of pure DBT are less dense than those of the **P**2_1_/*n* polymorph and appear to be less stable at all temperatures between 25 °C and the melting point. Because preparing and handling the new **Pnma** form of pure DBT were difficult, we were not able to characterize it by differential scanning calorimetry.

Successful generation of the new metastable **Pnma** form of DBT by templated heteroseeding encouraged us to attempt to make the unknown **P**2_1_/*n* forms of DBF, FLU, and CBZ by similar methods, using **P**2_1_/*n* crystals of DBT as seeds. In fact, crystallization of DBF, FLU, and CBZ could be induced by condensing their vapors onto the surface of crystals of DBT, but only the known **Pnma** forms were produced. Templated heteroseeding is a promising way to create new solid forms; however, our observations suggest that its effectiveness may be correlated with the ability of the compound of interest to form mixed crystals with the component of the seeds. If so, the likelihood of successful heteroseeding can be assessed by the computational approach we have used.

## Conclusions

In mixed crystallization, variable ratios of structurally analogous components can occupy sites in the lattice at random. The phenomenon remains poorly explored, even though it creates significant problems and opportunities. For example, it compromises the utility of crystallization as a general method of purification; at the same time, however, mixed crystals are valuable because their properties can be adjusted in increments, simply by altering the composition. For these reasons and others, it is important to develop a better understanding of when different compounds can form mixed crystals, what ratios of components can be accommodated, and how mixed crystals can be put to use. When closely similar compounds crystallize isostructurally, mixed crystals can typically be formed in all proportions. However, when compounds are not known to crystallize isostructurally, the feasibility of mixed crystallization and the range of accessible compositions are not governed by simple rules.

To begin to develop clear guidelines, we have used single-crystal X-ray diffraction, computational methods, and other approaches to study mixed crystallizations within a set of four structural analogues: DBT, DBF, CBZ, and FLU. The normal **Pnma** structures of DBF, CBZ, and FLU can accommodate large amounts of DBT, whereas the preferred **P**2_1_/*n* structure of DBT excludes significant amounts of the other compounds. Our computational modeling, in which we evaluate the energies of supercells created from the reported **Pnma** and **P**2_1_/*n* structures by randomly swapping the components, shows that the complex behavior of mixed crystals can be predicted successfully. For the DBT:DBF system, our computational studies show that the free energy of the observed **Pnma** mixed crystals varies smoothly with composition and is insensitive to how molecules of DBT and DBF are placed within the supercell. In contrast, the calculated free energy of the unobserved **P**2_1_/*n* mixed crystals changes much more steeply with composition, and rearrangement to different structures can occur, depending on how the components are arranged in the supercell. Our approach thereby allows host structures that accommodate the formation of mixed crystals to be distinguished from those that do not, provides a way to compare the free energies of alternative mixed crystals, and reveals how the free energies of mixed crystals differ from those of the pure components. In such ways, our method provides access to a detailed understanding of mixed crystallization, when adequate numbers of supercell configurations are sampled.

Further studies of this type, in which experimental and computational methods are used in tandem, can be expected to clarify the rules governing mixed crystallization, despite the complexity of the phenomenon. New understanding arising from this work promises to lead to the creation of useful mixed crystals with compositions and properties that vary continuously over wide ranges, even when the individual components do not prefer to crystallize isostructurally.

## Experimental Section

DBT, DBF, FLU, and CBZ were purchased from commercial suppliers and used without further purification. Raman spectra were recorded using a Renishaw inVia Reflex spectrometer, with light from a 785 nm 200 mW laser passing through an 1800 l/mm grating. Data were acquired using a 5× objective lens with a numerical aperture of 0.12, a 50 μm monochromator slit, and a 25 μm confocal slit, resulting in a spot size of about 15 μm. The exposure time was 1 sec, and the spectral range used was 440–1110 cm^–1^, with a nominal spectral resolution of 1 cm^–1^ per pixel. Calibration was done with a Si reference sample. Raman mapping experiments were carried out in a similar way. Individual measurements were spaced 10 μm apart in a rectangular grid spanning 640 × 980 μm^2^, giving a total of 6435 spectra. WiRE 5.2 software was used to remove the effect of cosmic rays and to filter noise. Integration of signals in the ranges 690–720 and 730–760 cm^–1^ was carried out to quantify the relative amounts of DBT and DBF, respectively. The resulting map represents the ratio of these integrated peaks (DBT/DBF) at each position. Differential scanning calorimetry was performed using a PerkinElmer DSC 6000 calorimeter with manually crimped Al pans containing samples weighing approximately 3 mg.

### Preparation of Pure and Mixed Crystals of DBT, DBF, FLU, and CBZ

The pure compound or a mixture with a defined ratio of components was dissolved in boiling MeOH (about 5 mL per gram of solid), and the solution was allowed to cool slowly to 25 °C. The vessel was sealed with Al foil, a small hole was made in the foil, and crystallization was induced by letting the solvent evaporate slowly during a week.

### Preparation of **Pnma** Crystals of DBT by Sublimation

DBT (about 10 mg) was placed in a vial and warmed near its melting point. A **Pnma** seed crystal (pure DBF, pure FLU, pure CBZ, or mixtures of DBT and DBF) was lodged in the tip of a glass pipet. The pipet was placed above the warmed sample of DBT and connected to gentle aspiration to draw vapors over the seed. After a few minutes of sublimation, the pipet was withdrawn, and **Pnma** crystals of pure DBT could be collected from the tip.

## Supporting Information Available

Additional crystallographic information (including thermal atomic displacement parameter plots) and a detailed description of computational modeling (PDF)
