# Supplementary material for: Seeking Rules Governing Mixed Molecular Crystallization
Source: Cryst Growth Des. 2022 Dec 15;23(1):273–88. doi: 10.1021/acs.cgd.2c00992 (PMC9817076; doi:10.1021/acs.cgd.2c00992)
Supplement: Supplementary file 1 — cg2c00992_si_001.pdf [file cg2c00992_si_001.pdf]

Supporting Information

Seeking Rules Governing Mixed Molecular Crystallization

Norbert M. Villeneuve,[†] Joshua Dickman,[‡] Thierry Maris,[†]

Graeme M. Day,*[,‡] and James D. Wuest*[,†]

[†]*Département de Chimie, Université de Montréal, Montréal, Québec H2V 0B3 Canada*

[‡]*School of Chemistry, University of Southampton, University Road, Southampton SO17 1BJ United Kingdom*

*Authors to whom correspondence may be addressed

Email: *james.d.wuest@umontreal, g.m.day@soton.ac.uk*

Contents **Page**

I.	Additional Crystallographic Information	S3
II.	Thermal Atomic Displacement Parameter Plots	S6
III.	Indexation of Crystal Faces	S8
IV.	Detailed Description of Computational Modeling	S9
V.	References	S25

I. Additional Crystallographic Information

Data for the mixed crystals DBT:DBF 0.79:0.21, DBT:CBZ 0.62:0.38, and DBT:FLU 0.22:0.78 were collected on a Bruker Venture Kappa-geometry diffractometer equipped with a gallium liquid metal-jet source (GaKα radiation, λ = 1.34139 Å), a Photon 100 CMOS detector, and Helios MX mirror optics. For all other compounds, diffraction data were measured on a Bruker Smart three-circle diffractometer equipped with an Incoatec Microfocus Source (IμS) delivering CuKα radiation (λ = 1.54178 Å) and an APEX II CCD detector. The data were integrated with *SAINT* V8.40B,[1] and a multi-scan absorption correction using *SADABS*[2] was applied. Structures were solved using the intrinsic phasing routine implemented in *SHELXT*[3] and were refined using least-square minimization with *SHELXL*[4] within the graphical user interface of *OLEX2*.[5] All non-hydrogen atoms were refined with anisotropic displacement parameters. For all the mixed crystals, hydrogen atoms were refined using the standard riding model of *SHELXL*, whereas the hydrogen atoms for the *Pnma* polymorph of DBT were located from the Fourier difference map and refined isotropically.

For all the DBT:DBF mixed crystals, the only restraint that was introduced in the refinement was applied on the thermal atomic displacement parameters of the atoms in the vicinity of the atom of sulfur of DBT (SIMU restraints applied on C1, S1, and O1). The same kind of restraints were applied for the disordered atoms in all the other mixed crystal structures, in addition to the following:

1) For the DBT:FLU mixed crystal (0.22:0.78), a FLAT restraint was applied for the whole DBT molecule.
2) For the ternary DBT:DBF:FLU mixed crystal (0.32:0.23:0.45), the SIMU restraints on the atomic thermal displacement parameters involved C1, S1, O1, and the extra C7 atom from FLU.
3) The structure of the mixed crystal DBF:FLU 0.31:0.69 needed additional restraints, as this structure displays the same statistical disorder found for crystals of pure DBF (two orientations flipped by 180 degrees with relative occupation of 0.90:0.10).[6,7] The refinement involved restraints on C-O distances, and the two disordered oxygen atoms O1A and O1B were constrained to have the same ADP to stabilize the refinement.

More details related these refinements can be found in the deposited CIF data, and additional crystallographic information about mixed crystals other than those described in Table 4 is provided below in Table S1.

Table S1. Selected Crystallographic Data for Mixed Crystals of DBT with Components Other Than DBF

compound<br>component ratio	DBT:CBZ<br>0.62:0.38	DBT:FLU<br>0.22:0.78	DBF:FLU<br>0.31:0.69	DBT:DBF:FLU<br>0.32:0.23:0.45
CSD refcode	2195721	2195726	2195720	2195722
description	colorless plates	colorless plates	colorless plates	colorless plates
crystal syst	orthorhombic	orthorhombic	orthorhombic	orthorhombic
space group	*Pnma*	*Pnma*	*Pnma*	*Pnma*
T (K)	150	150	100	100

radiation/λ (Å)	GaKα/ 1.34139	GaKα/ 1.34139	CuKα/ 1.54178	CuKα/ 1.54178
a (Å)	7.9749(3)	8.2901(2)	8.0978(3)	8.0589(2)
b (Å)	18.9409(6)	18.8144(5)	18.8577(8)	18.8454(4)
c (Å)	5.7813(2)	5.7082(1)	5.7063(2)	5.7503(1)
α (deg)	90	90	90	90
β (deg)	90	90	90	90
γ (deg)	90	90	90	90
V (Å^3)	873.28(5)	890.33(4)	871.39(6)	873.32(3)
Z	4	4	4	4
Z'	0.5	0.5	0.5	0.5
ρ_{calc} (g · cm^{-3})	1.352	1.269	1.272	1.312
collected	8887	7825	10615	9399
R_{int}/R_σ	0.0402 / 0.0255	0.0308 / 0.0197	0.0236 / 0.0129	0.0186 / 0.0087
unique	1026	1047	871	879
observed	999	997	816	847
nb. parameters	68	80	99	76
R_1, $I > 2\sigma(I)$	0.0329	0.0370	0.0558	0.0347
wR_2, $I > 2\sigma(I)$	0.0871	0.0989	0.1550	0.0936
R_1, all data	0.0333	0.0381	0.0581	0.0355
wR_2, all data	0.0874	0.1001	0.1578	0.0945
GoF	1.074	1.079	1.033	1.082
max/min residual electron density (e/Å^3)	0.222 /-0.144	0.221/-0.144	0.350 /-0.400	0.303/-0.251

II. Thermal Atomic Displacement Parameter Plots

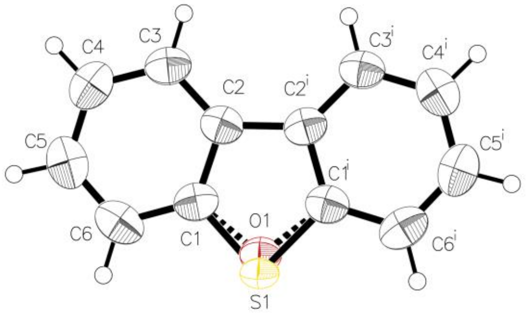

DBT:DBF 0.23:0.77

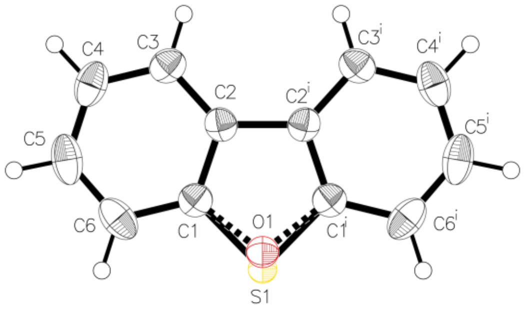

DBT:DBF 0.46:0.54

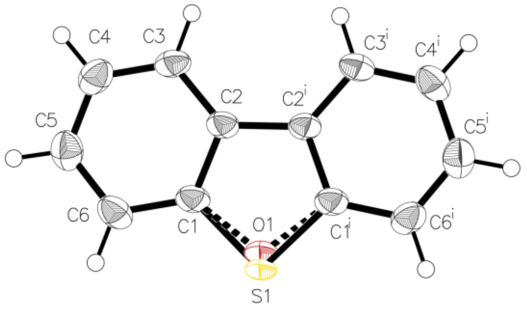

DBT:DBF 0.59:0.41

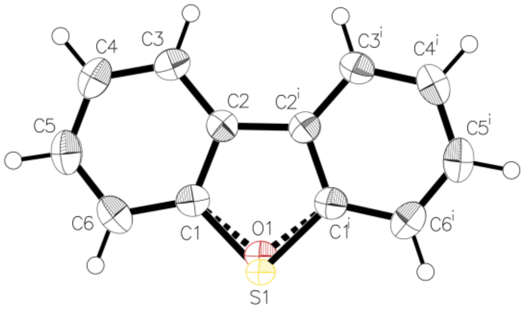

DBT:DBF 0.73:0.27

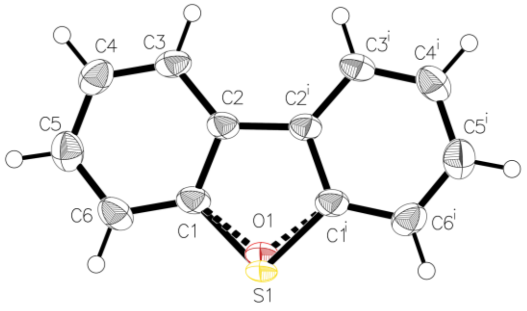

DBT:DBF 0.79:0.21

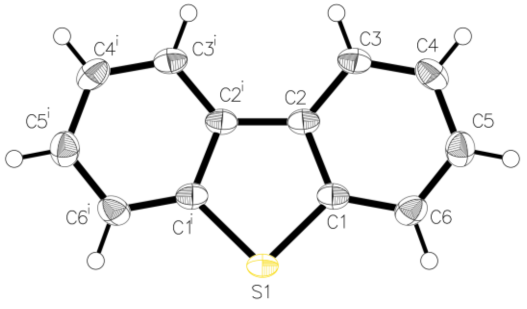

DBT (1) *Pnma* polymorph

Figure S1. Thermal atomic displacement ellipsoid plots of the structures of DBT/DBF mixed crystals and the *Pnma* polymorph of DBT with the atom numbering schemes. The ellipsoids of non-hydrogen atoms are drawn at the 50% probability level, and hydrogen atoms are represented by a sphere of arbitrary size. Symmetry Codes: (i) = x, 1/2-y, z.

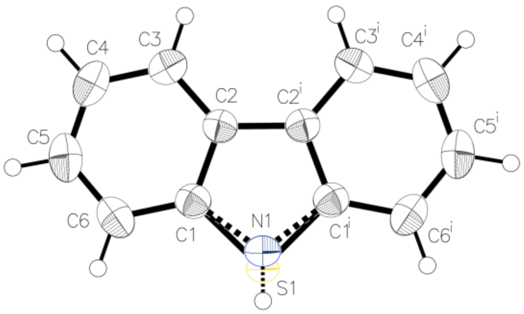

DBT:CBZ 0.62:0.38

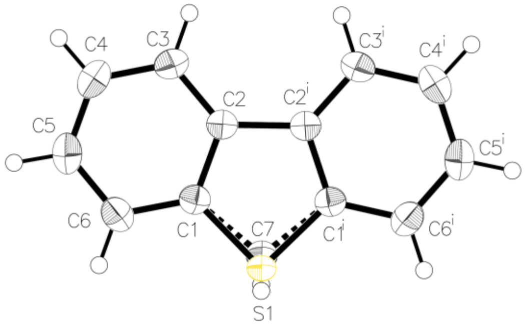

DBT:FLU 0.22:0.78

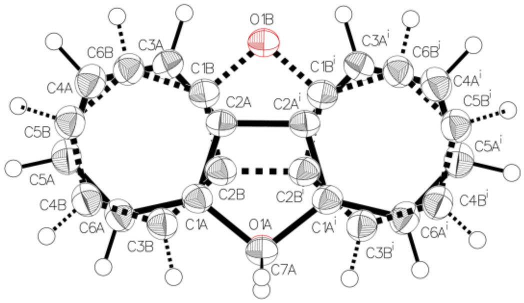

DBF:FLU 0.31:0.69

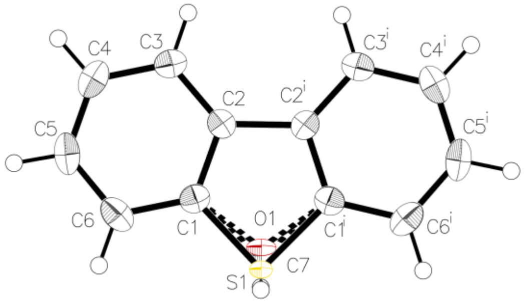

DBT:DBF:FLU 0.32:0.23:0.45

Figure S2. Thermal atomic displacement ellipsoid plots of the structures of other mixed crystals with the atom numbering schemes. The ellipsoids of non-hydrogen atoms are drawn at the 50% probability level, and hydrogen atoms are represented by a sphere of arbitrary size. Symmetry Codes: (i) = x, 1/2-y, z.

III. Indexation of Crystal Faces

DBT

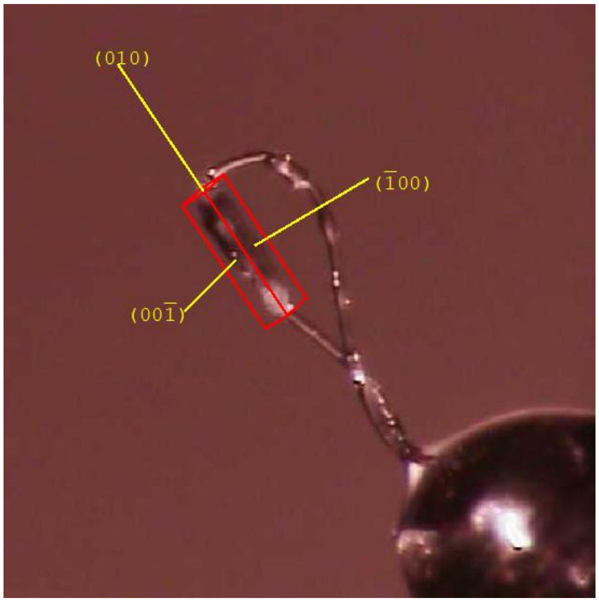

FACE	0	0	-1	0.067
FACE	0	0	1	0.042
FACE	0	-1	0	0.122
FACE	0	1	0	0.081
FACE	-1	0	0	0.052
FACE	1	0	0	0.056

DBF

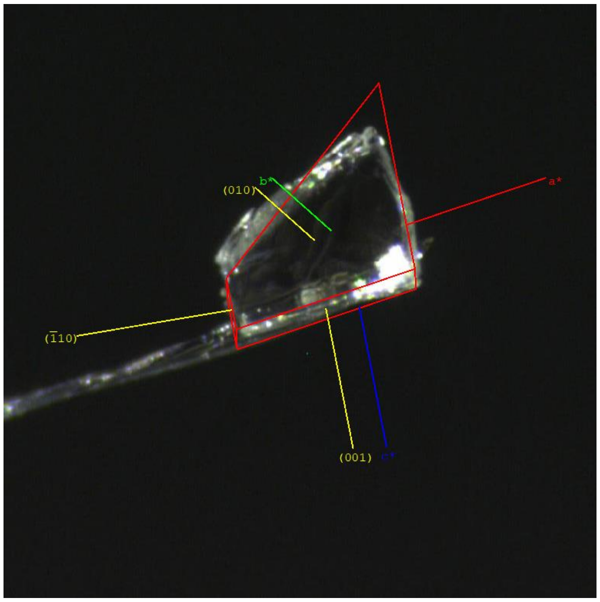

FACE	-1	1	0	0.241
FACE	-1	0	-1	0.130
FACE	-1	1	-1	0.134
FACE	0	0	1	0.136
FACE	0	1	0	0.084
FACE	0	-1	0	0.001
FACE	1	-1	0	0.111

IV. Detailed Description of Computational Modeling

Crystal Structure Prediction (CSP). Trial crystal-packing arrangements were constructed by mapping structural degrees of freedom, such as lattice parameters and molecular positions, from a low-discrepancy sequence of vectors generated by the method of Sobol.[8] The trial structures were then subjected to SAT-expansion (Separating Axis Theorem) to prevent molecular overlap, followed by lattice-energy minimization with DMACRYS software[9] to locate the closest energy minimum on the local potential energy surface. Structures were generated in the 10 most common space groups for small organic molecules, using the FIT exp-6 atom-atom potential combined with atomic multipoles from a distributed multipole analysis (DMA)[10] of PBE0/6-311G** charge density. Multipoles up to hexadecapole were included on all atoms.

For the generated landscape of each species, structures within the lowest 7 $kJ \cdot mol^{-1}$ energy window were compared with the experimental crystal structures retrieved from the Cambridge Structural Database (CSD), in order to locate the CSP-generated structure matching existing experimental data. The structure that matched the CSD entry of its species with the lowest root-mean squared deviation of atomic positions in a 30-molecule cluster of molecules taken from the crystal structures ($RMSD_{30}$) was used in later steps to construct supercells for mixed-crystal models. Larger versions of the CSP landscape plots in the main text (Figure 4) are shown below in Figures S3–S6, with additional information provided for DBF (Figure S4).

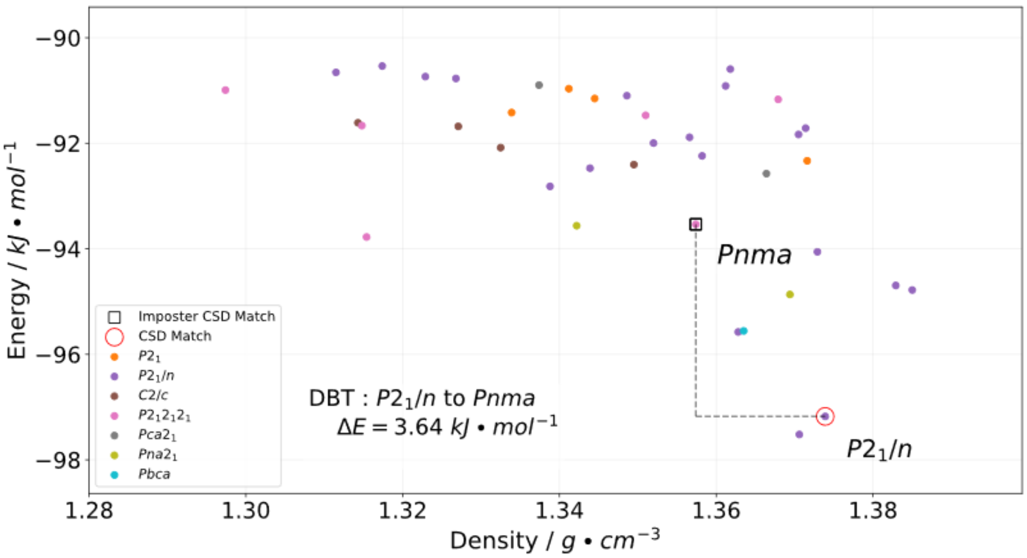

Figure S3. Predicted crystal-structure landscape for DBT. Structures are labeled according to the space group in which they were located during the CSP search. The structure of second-lowest energy matches experimental data for the known $P2_1/n$ form. The marked *Pnma* structure matches experimental data extracted from the CSD for DBF, FLU, and CBZ.

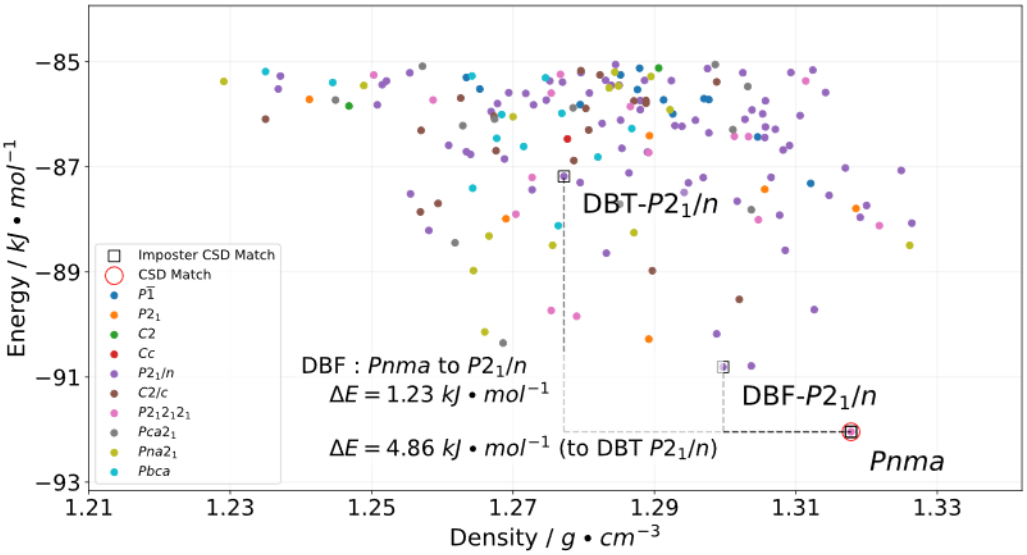

Figure S4. Predicted crystal-structure landscape for DBF. Structures are labeled according to the space group in which they were located during the CSP search. The global minimum CSP structure matches experimental data for the known *Pnma* form of DBF (CSD reference code DBZFUR) and also matches the packing of the known crystal structures of FLU and CBZ. Optimization of the $P2_1/n$ mixed-crystal supercell of DBF gave a structure matching a CSP $P2_1/n$ structure on this landscape. A higher-energy $P2_1/n$ structure, located 4.86 kJ · mol^{-1} above the global minimum, matches experimental data for DBZTHP01.

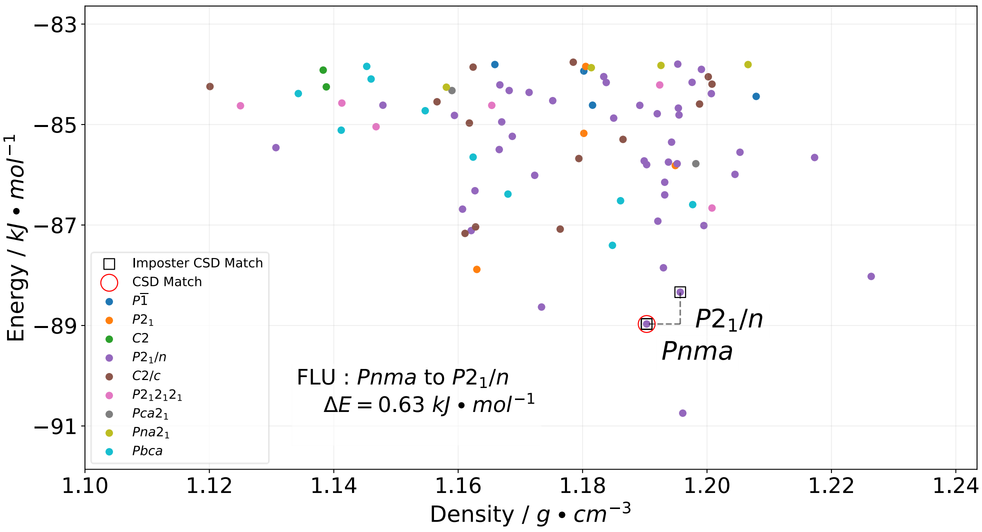

Figure S5. Predicted crystal-structure landscape for FLU. Structures are labeled according to the space group in which they were located during the CSP search. The structure of second-lowest energy matches experimental data for the *Pnma* form, which is also favored by DBF and CBZ. The marked $P2_1/n$ form matches experimental data for DBT.

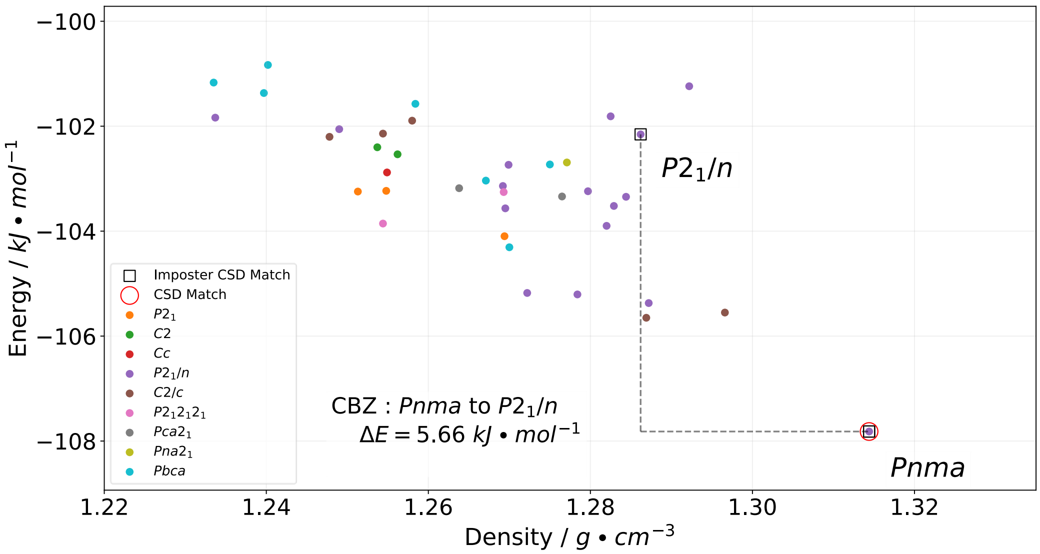

Figure S6. Predicted crystal-structure landscape for CBZ. Structures are labeled according to the space group in which they were located during the CSP search. The global minimum structure matches experimental data for the *Pnma* form, which is also favored by FLU and DBF. The marked $P2_1/n$ structure matches CSD data for DBT.

Construction of Mixed Crystals. Supercells containing 32 molecules were generated from the known DBF and DBT unit cells to sample a wide range of compositions, from pure to fully substituted, in both *Pnma* and $P2_1/n$ forms. Overly anisotropic structures can create problems during optimization, so before the construction of supercells, the host unit cell was subjected to Niggli unit-cell standardization. The standardized cell was then used as a repeating unit to construct 2 × 2 × 2 supercells of the packing arrangement of the host species, from which a set of molecules was randomly chosen to be replaced with the imposter species.

Because molecular substitutions are chosen at random before optimization of the mixed-crystal supercell, multiple versions of each supercell with different substitution patterns were generated. This was necessary because two mixed crystals of the same composition but with different patterns of substitution may give rise to different packing arrangements post-optimization. To ensure reproducibility, random selection of the positions of substitution positions was performed using Python *random* seeds.

This replacement results in a mixed crystal of host and imposter in the packing arrangement of the host. To simulate the packing arrangement of the mixed crystal, including molecular replacement, the structure was reoptimized to the nearest energy minimum using DMACRYS via CSPy (FIT potential, PBE0 functional, and the cc-pVTZ basis). DMACRYS requires that multipoles describing each molecular component in a crystal must be optimized, so multipoles from the CSP of the host species and the separate DMACRYS processing of the imposter were joined in a single file.

Configurational entropy can be estimated (1) by calculating the number of possible configurations (*W*) with N molecules, where N_B molecules have been swapped and N_A are unswapped ($N = N_A + N_B$) and (2) by using the following equation:

$$S = k \, lnW \qquad W = \frac{(N_A + N_B)!}{N_A! N_B!}$$

This assumes that the energy values of different states at a given composition are similar. This is appropriate for *Pnma* mixed crystals of DBT and DBF, which follow a single path in energy as

composition changes. The $P2_1/n$ case, where multiple energy pathways appear, depending on the patterns of substitution, should be examined using a method which accounts for varying energy states.

We tested multiple patterns of substitution at each composition, so that the estimation of configurational entropy and its consequences can be adapted to include more information about the relative probabilities of each energy state. With the number of tested patterns of substitution that were successfully optimized (N_{trials}) and their energies (E_N), the equation takes the following form:

$$S = \frac{-k_B W}{N_{trials}} \sum_{i=1}^{N_{trials}} P_i \ln P_i$$

$$P_i = \frac{\exp(-\beta E)}{\sum \exp(-\beta E)} \qquad \beta = {}^{1}/_{k_B N_A T}$$

K_B = Boltzmann constant (1.38×10^{-23} $m^2 \cdot kg \cdot s^{-2} \cdot K^{-1}$)

N_A = Avogadro's number (6.02×10^{23})

With these equations, probabilities can be assigned to each energy state, with lower energies being preferred. The impact of having higher energy states can then be examined, but in cases where there are far more low-energy states than high, such as the $P2_1/n$ mixed crystals of DBT and DBF, the result will not differ much in comparison to the $S = k \cdot lnW$ approach.

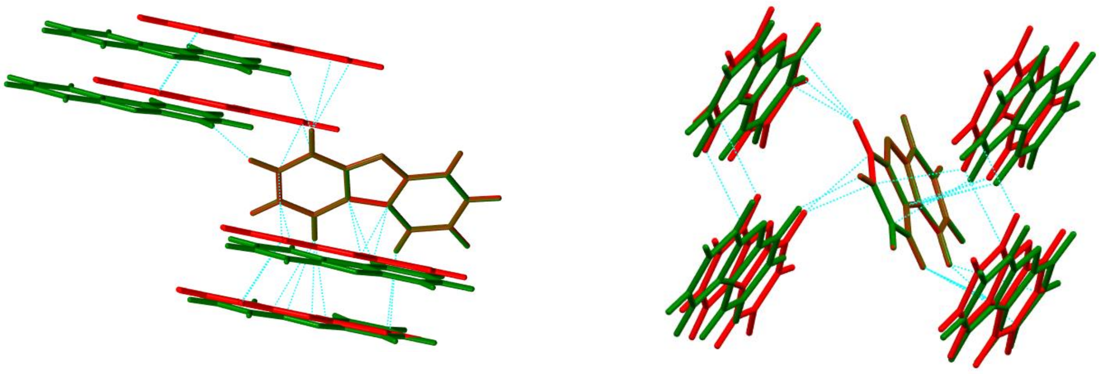

**DBF** *P*2_1/*n* **-** Low Energy *(opt_DBZFUR-QR-14-3076-3)*	
**DBF** *P*2_1/*n* **-** High Energy *(opt_DBZFUR-QR-14-11258-3)*	

Figure S7. Comparison of predicted high-energy and low-energy $P2_1/n$ structures of DBF to show the differences in packing. Green corresponds to the lower-energy structure, and red shows the higher-energy form.

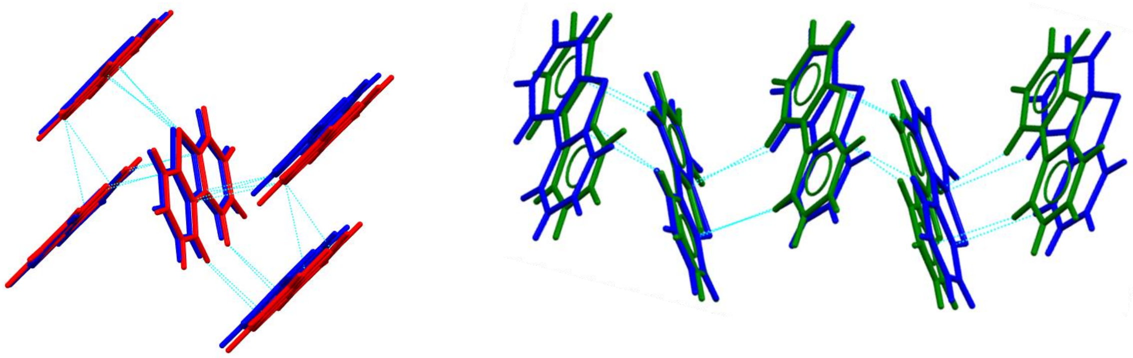

a **b**

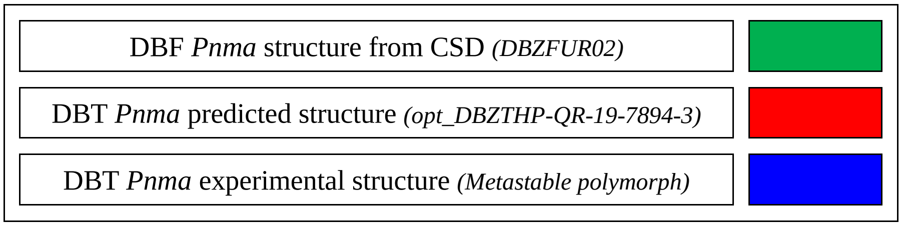

Figure S8. Comparisons of the observed *Pnma* structure of DBT. (a) Comparison with the predicted *Pnma* structure of DBT. (b) Comparison with the reported *Pnma* structure of DBF.

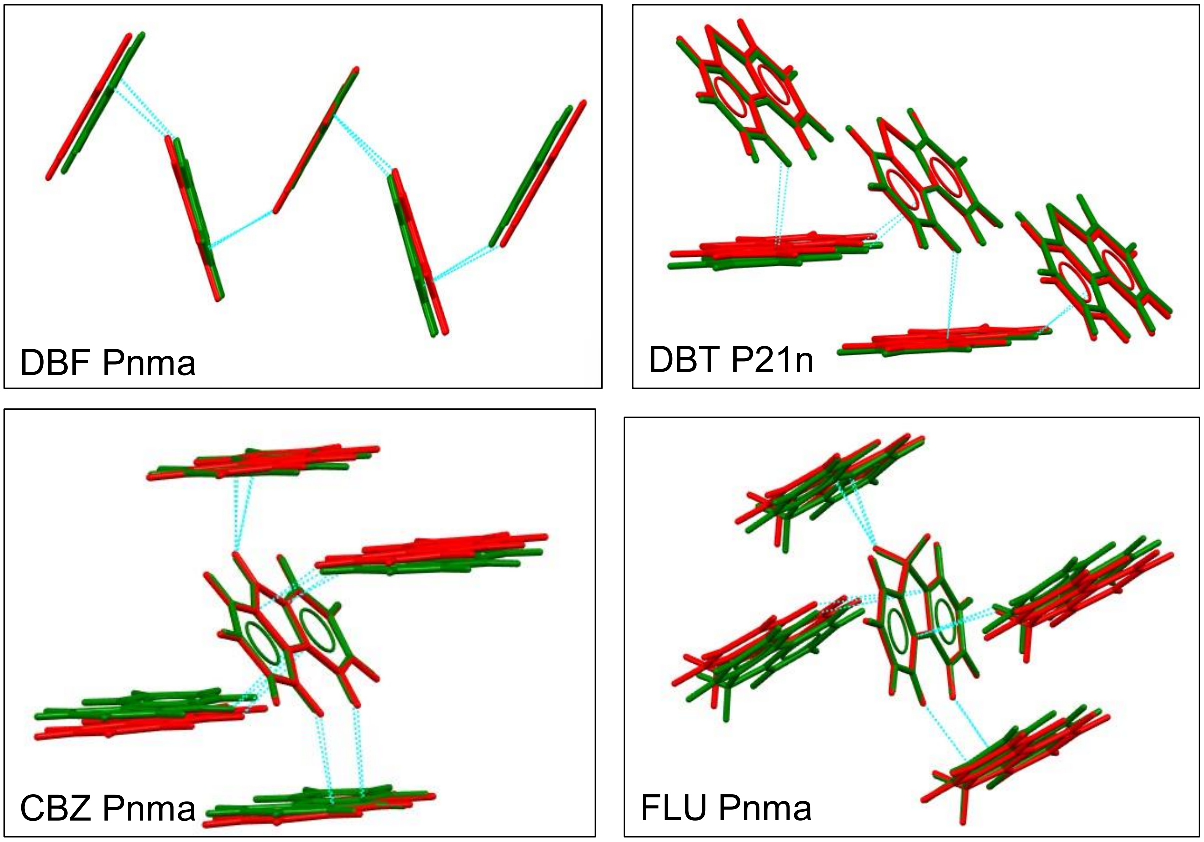

Figure S9. Comparison of both CSP and CSD structures of DBF, DBT, CBZ, and FLU to show the difference in packing between simulation and experiment for each species. Green corresponds to the CSD entry, and red shows the CSP-generated structure.

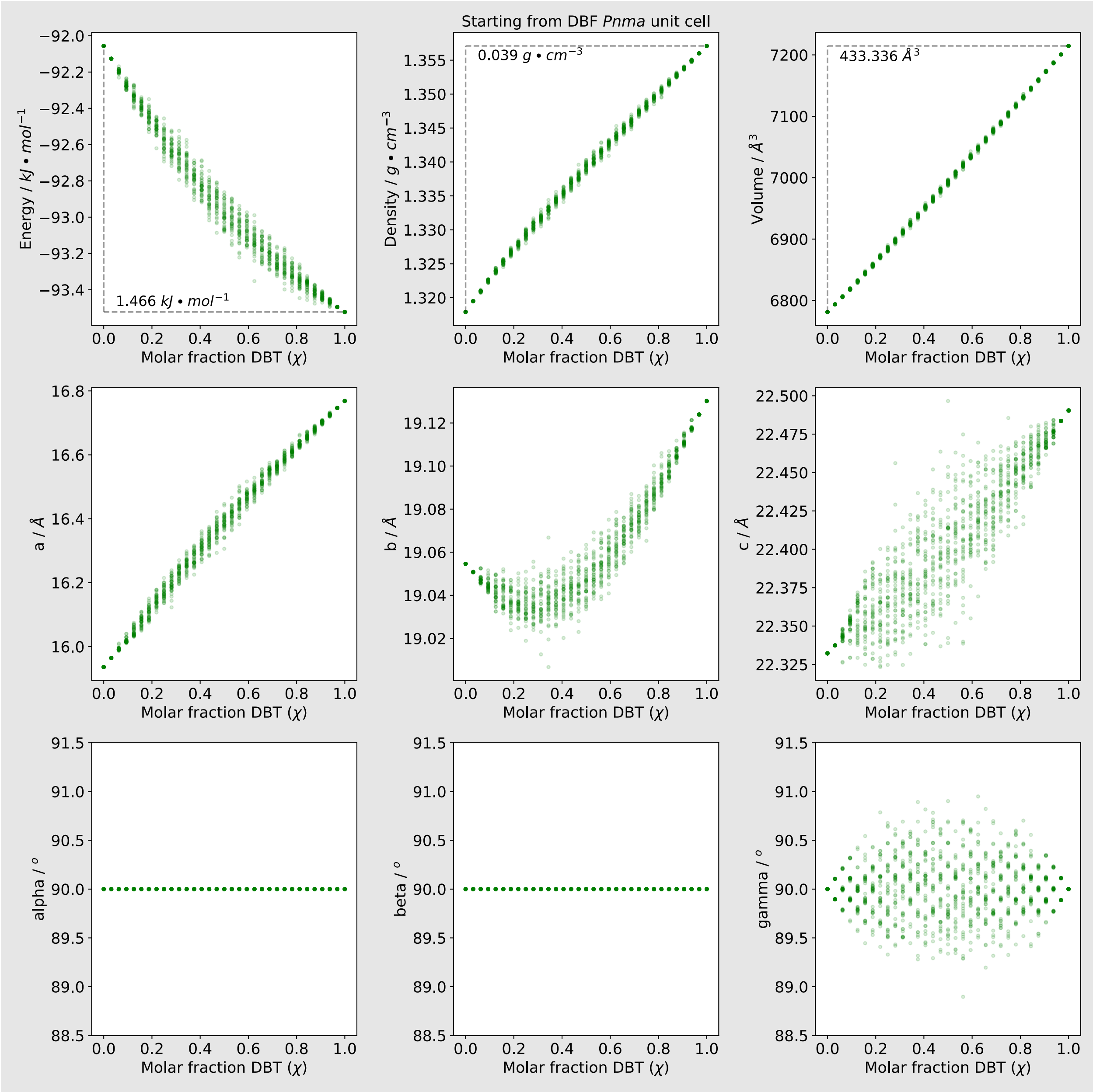

Figure S10. Plot showing the variation in lattice parameters as the composition of *Pnma* mixed-crystals of DBT and DBF changes. Each point corresponds to a different mixed-crystal model with minimized lattice energy, and 40 configurations were built for each composition.

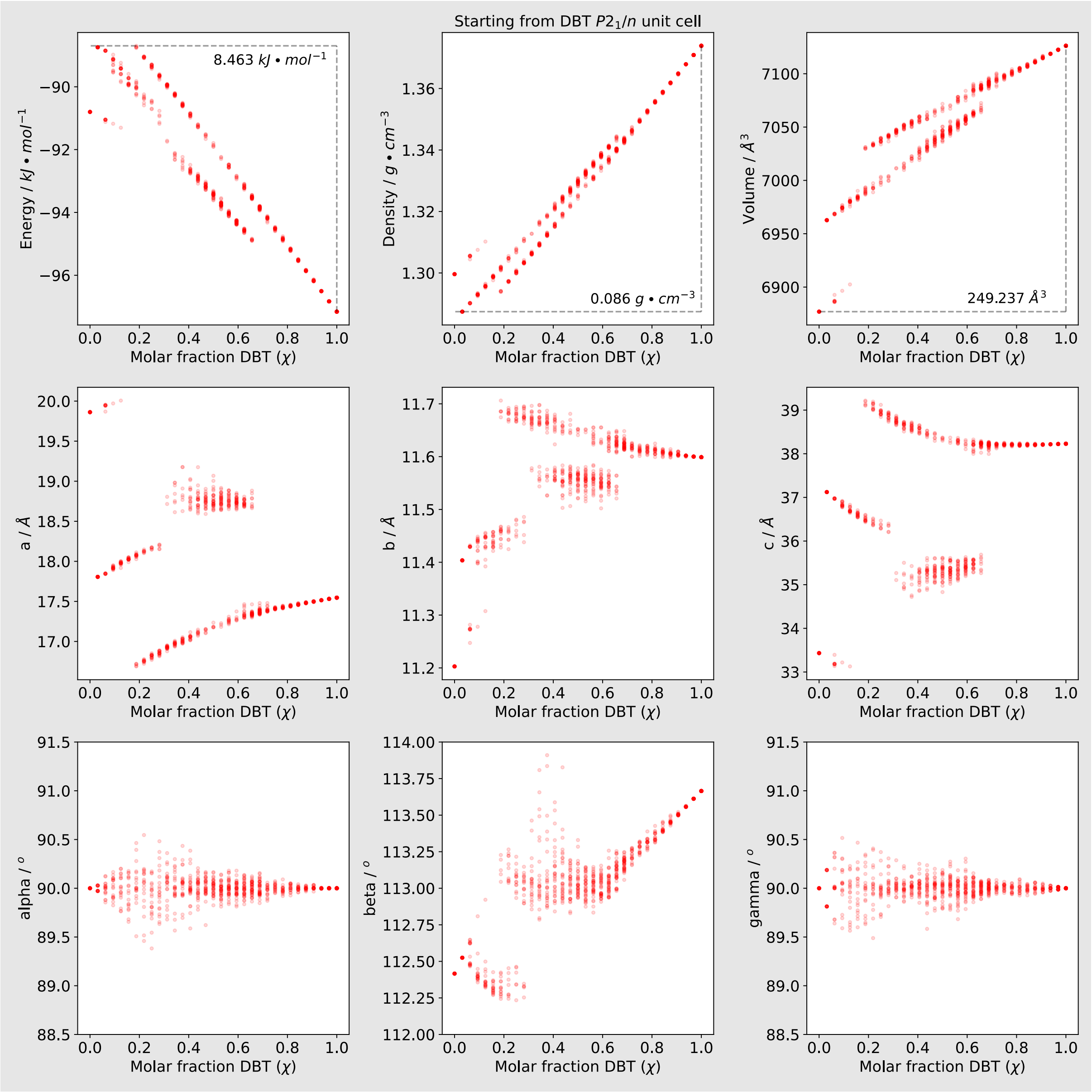

Figure S11. Plot showing the variation in lattice parameters as the composition of $P2_1/n$ mixed-crystal supercells of DBT and DBF changes using the 32-molecule ($2a \times 2b \times 2c$) supercell model. Each point corresponds to a different mixed-crystal model with minimized lattice energy, and 40 configurations were built for each composition.

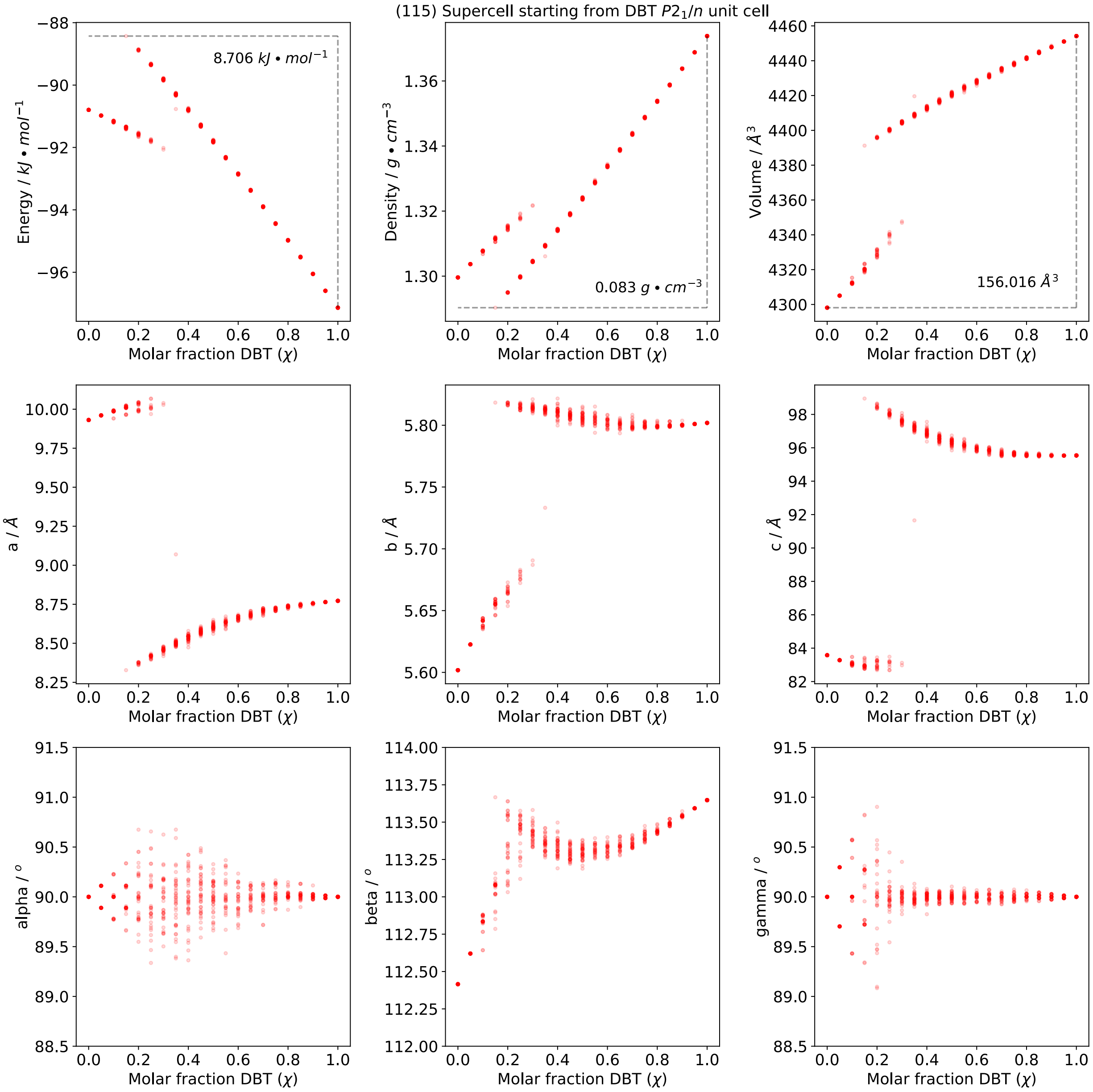

Figure S12. Plot showing the variation in lattice parameters as the composition of $P2_1/n$ mixed-crystal supercells of DBT and DBF changes using the 20-molecule ($a \times b \times 5c$) supercell model. Each point corresponds to a different mixed-crystal model with minimized lattice energy, and 40 configurations were built for each composition.

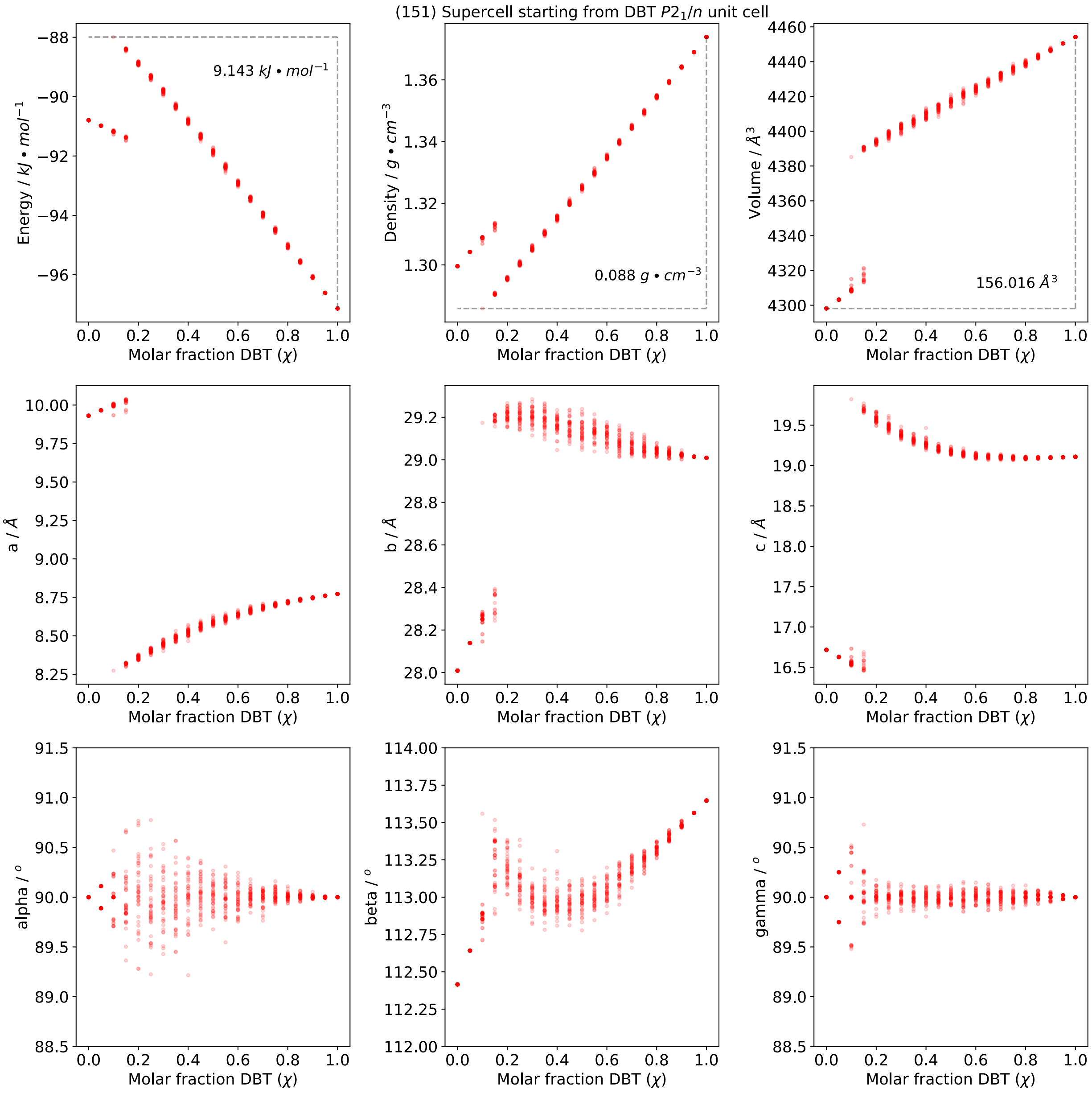

Figure S13. Plot showing the variation in lattice parameters as the composition of $P2_1/n$ mixed-crystal supercells of DBT and DBF changes using the 20-molecule ($a \times 5b \times c$) supercell model. Each point corresponds to a different mixed-crystal model with minimized lattice energy, and 40 configurations were built for each composition.

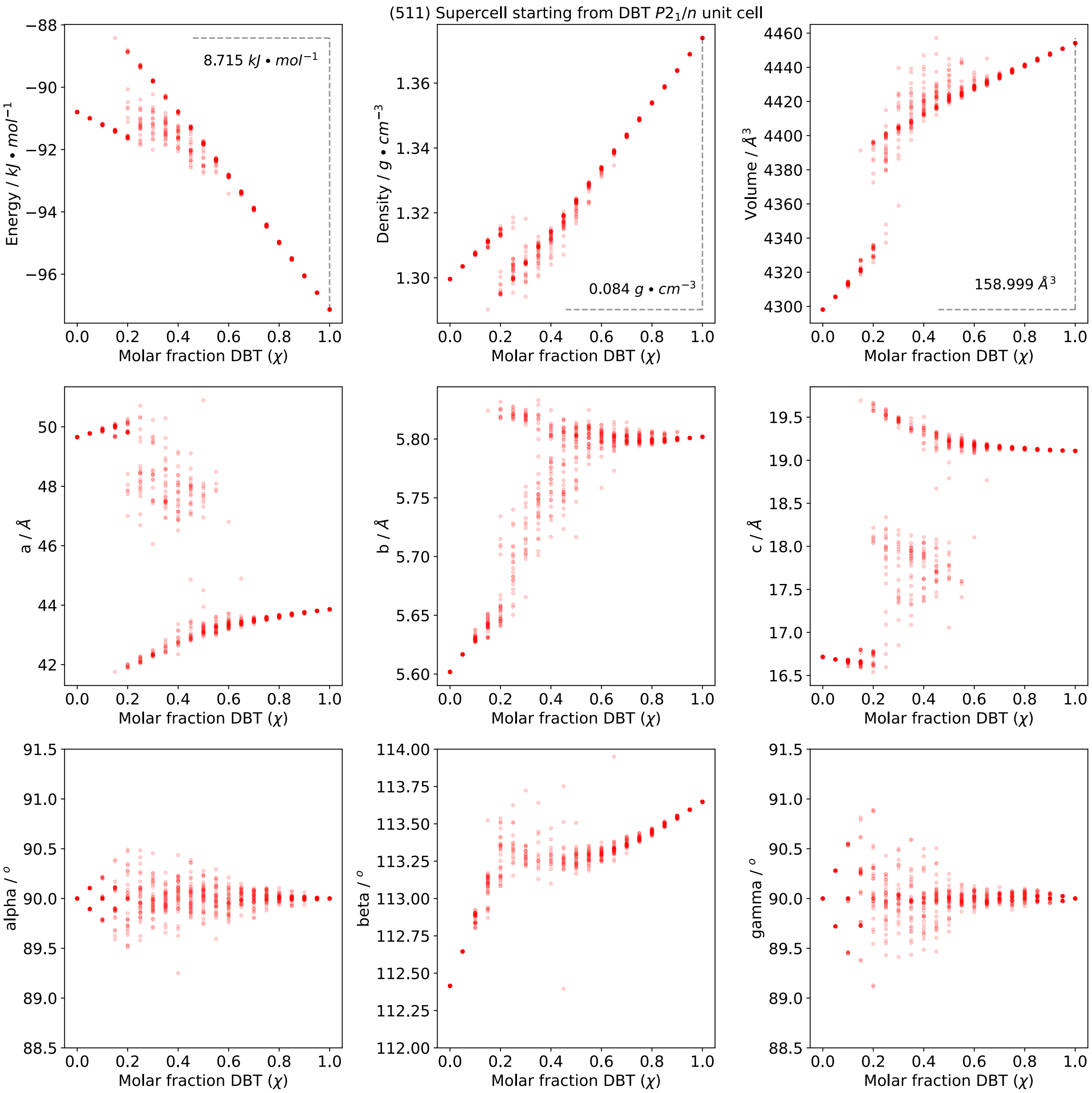

Figure S14. Plot showing the variation in lattice parameters as the composition of $P2_1/n$ mixed-crystal supercells of DBT and DBF changes using the 20-molecule ($5a \times b \times c$) supercell model..

Each point corresponds to a different mixed-crystal model with minimized lattice energy, and 40 configurations were built for each composition.

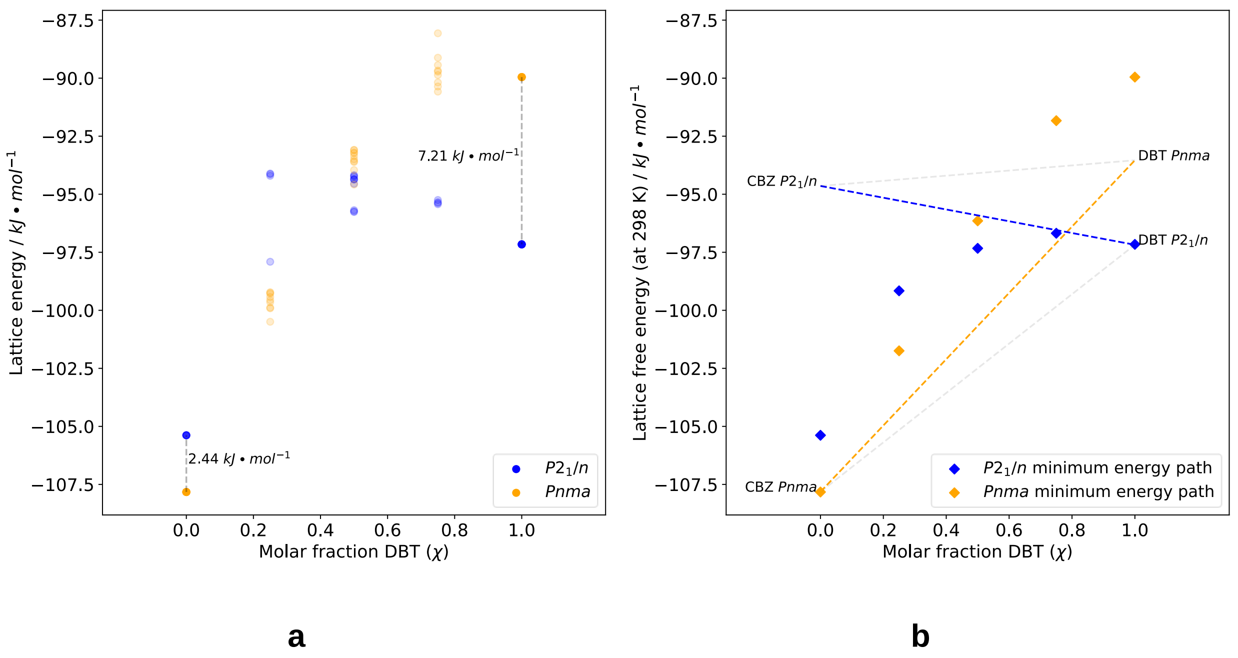

Figure S15. (a) Lattice energy of mixed crystals of DBT and CBZ initiated from their known *P*2$_1$/*n* and *Pnma* packing arrangements. (b) Lowest free energy pathways of simulated mixed crystals of DBT and CBZ at 298 K, with solid lines denoting the energy of the pure components.

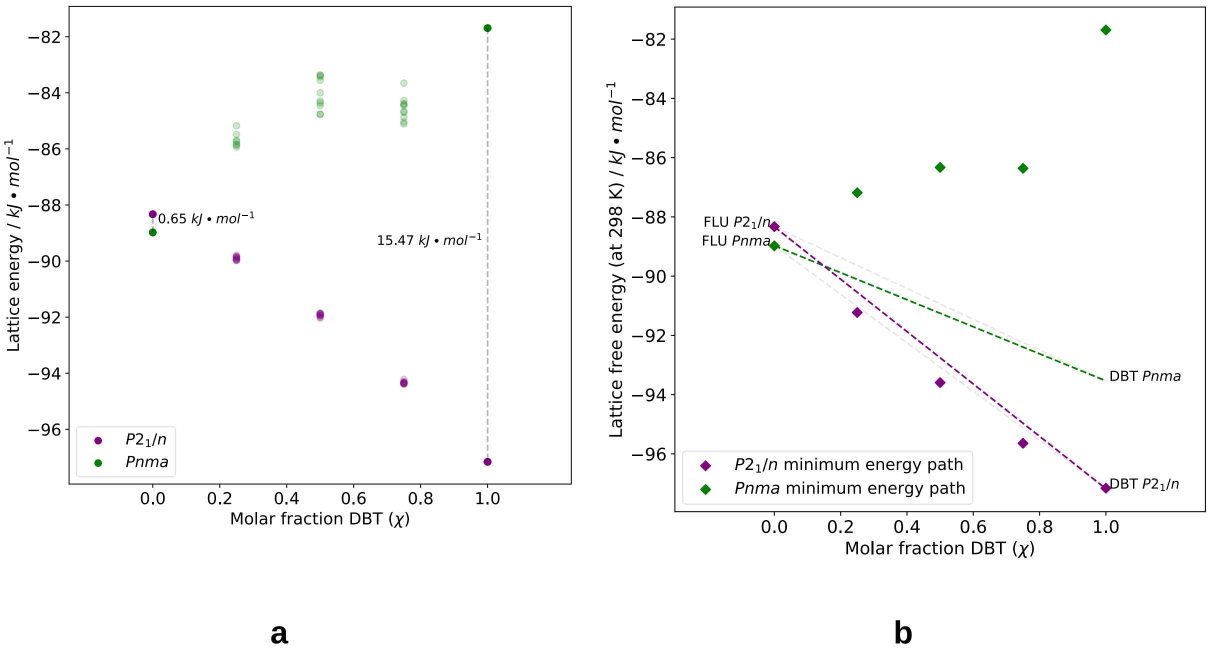

Figure S16. (a) Lattice energy of mixed crystals of DBT and FLU initiated from their known $P2_1/n$ and *Pnma* packing arrangements. (b) Lowest free energy pathways of simulated mixed crystals of DBT and FLU at 298 K, with solid lines denoting the energy of the pure components.

V. References

1. Bruker (2020). *APEX3* and *SAINT*, Bruker AXS Inc., Madison, Wisconsin, USA.
2. Krause, L.; Herbst-Irmer, R.; Sheldrick, G. M.; Stalke, D. Comparison of Silver and Molybdenum Microfocus X-Ray Sources for Single-Crystal Structure Determination. *J. Appl. Cryst*. **2015**, *48*, 3–10.
3. Sheldrick, G. M. *SHELXT* – Integrated Space-Group and Crystal-Structure Determination. *Acta Crystallogr*. **2015**, *A71*, 3–8.
4. Sheldrick, G. M. Crystal Structure Refinement with *SHELXL*. *Acta Crystallogr*. **2015**, *C71*, 3–8.
5. Dolomanov, O. V.; Bourhis, L. J.; Gildea, R. J.; Howard, J. A. K.; Puschmann, H. *OLEX2*: A Complete Structure Solution, Refinement and Analysis Program. *J. Appl. Crystallogr*. **2009**, *42*, 339–341.
6. Fitzgerald, L. J.; Gallucci, J. C.; Gerkin, R. E. Structure of Dibenzofuran-d_8, $C_{12}D_8O$, at 173 K. *Acta Crystallogr., Sect. C: Cryst. Struct. Commun.* **1993**, *49*, 398−400.
7. Reppart, W. J.; Gallucci, J. C.; Lundstedt, A. P.; Gerkin, R. E. Order and Disorder in the Structure of Dibenzofuran, $C_{12}H_8O$. *Acta Crystallogr., Sect. C: Cryst. Struct. Commun.* **1984**, *40*, 1572−1576.
8. Sobol, I. M. On the Distribution of Points in a Cube and the Approximate Evaluation of Integrals. *Comput. Math. Math. Phys.* **1967**, *7*, 86–112.
9. Price, S. L.; Leslie, M.; Welch, G. W. A.; Habgood, M.; Price, L. S.; Karamertzanis, P. G.; Day, G. M. Modelling Organic Crystal Structures Using Distributed Multipole and Polarizability-Based Model Intermolecular Potentials. *Phys. Chem. Chem. Phys.* **2010**, *12*, 8478–8490.

10. Stone, A. J.; Alderton, M. Distributed Multipole Analysis. Methods and Applications. *Mol. Phys.* **2002**, *100*, 221–233.
